# Influence of Aluminum Distribution in Cu-MOR Systems on Methane-to-Methanol Conversion: A Combined Experimental and Theoretical Study

**DOI:** 10.1021/acs.jpcc.5c06045

**Published:** 2025-10-18

**Authors:** Peter N. Njoroge, Bjørn Gading Solemsli, Asanka Wijerathne, Izar Capel Berdiell, Agnieszka Seremak, Mario Chiesa, Yu-Kai Liao, Beatrice Garetto, Nishant Patel, Karoline Kvande, Elisa Borfecchia, Christopher Paolucci, Unni Olsbye, Pablo Beato, Stian Svelle, Sebastian Prodinger

**Affiliations:** † Centre for Materials Science and Nanotechnology (SMN), Department of Chemistry, 6305 University of Oslo, 1033 Blindern, Oslo 0315, Norway; ‡ Department of Chemical Engineering, 2358 University of Virginia, Charlottesville, Virginia 22903, United States; § Department of Chemical and Biomolecular Engineering, 1438 University of California, California, Berkeley 94720, United States; ∥ Department of Chemistry, NIS Centre and INSTM Reference Centre, 5193 University of Turin, Via P. Giuria 7, Turin 10125, Italy; ⊥ 87087 Topsoe A/S, Haldor Topsøes Allé 1, Kongens Lyngby 2800, Denmark

## Abstract

A series of copper-mordenite (MOR) samples of different provenances were investigated in the methane-to-methanol (MTM) reaction after preparing their copper-exchanged analogues. Noticeable activity improvements were observed when biasing the Al framework distribution of the confined side-pocket toward 12-ring openings (T2 and T4 enrichment) over 8-ring openings (T1 and T3), achieved by using K^+^ or Na^+^ in the synthesis gel, respectively. This was rationalized by performing a geometry optimization algorithm using density functional theory (DFT) simulations, which revealed distortions in the structure of the pores among different idealized zeolite models. From this, effects on the copper species were observed, as evidenced from both electron paramagnetic resonance (EPR) spectroscopy and *operando* X-ray absorption spectroscopy (XAS), which suggested varying monomeric [Cu]^2+^/[CuOH]^+^ concentrations with intrinsic copper reducibility differences. Monte Carlo simulations on selected MOR structures of the experimental series exposed dimeric structures with more acute Cu–O–Cu angles, thereby suggesting a more reactive system for Cu-MOR based on K^+^ in the synthesis gel, in line with the experimental finding. The combined insights from simulations, calculations, and experiments have enabled us to establish a synthesis-structure–activity relationship for mordenite in methane conversion, highlighting the reactive interplay between pore geometry and copper speciation.

## Graphical abstract

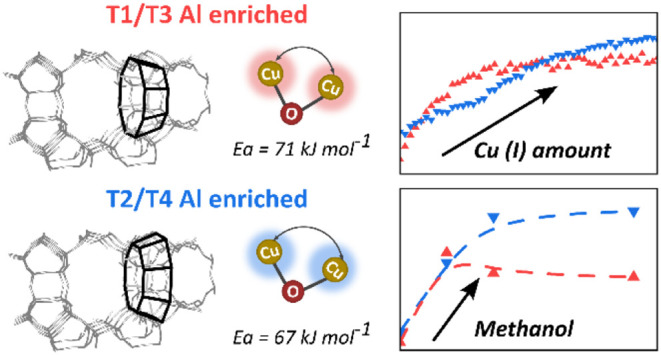

## Introduction

Inspired by methanotrophic enzymes like pMMO found in nature, where Cu single-atom sites facilitate C–H bond activation of methane under ambient conditions in the presence of oxygen,^1–3^ researchers have turned to the incorporation of copper into industrially applied catalysts. Zeolites, with their high nanoporosity, are ideal candidates to mimic this behavior, as they allow single-atom catalysts to be confined within the aluminosilicate network. This confinement leads to the modification of the active species’ redox properties, thus improving its activity.^4–6^ A three-step cyclic chemical process has been adapted to form methanol from methane and oxygen over transition metal-zeolites.^7–10^ Among the different zeolite topologies screened in the literature, mordenite (MOR) emerges with the highest activity toward the methane-to-methanol (MTM) reaction.^7,10–12^

MOR is classified as a large-pore zeolite with straight 12-ring channels (7.0 × 6.5 Å) and compressed 8-ring channels (5.7 × 2.6 Å) parallel to each other in the *c* direction (Figure 1).^11,13,14^ These two channels are interconnected by intercalated 8-rings, referred to as the side pocket. MOR has four unique T-sites that can be occupied by Al in the framework. Here, control over the Al bias (e.g., via the choice of structure-directing agent)^15^ can enact a unique feature, altering the aperture size of the pore system to be denominated either as small-port or large-port MOR.^16^ This feature was first seen as different variants of MOR were able to adsorb differing amounts of molecules larger than 4.2 Å (small-port MOR: less than 5 wt % benzene/toluene).^13,16^ Knorpp et al. reported a connection between small- and large-port MOR and its activity toward the MTM reaction^13^ with the large-port variant having a higher methanol yield. They argued that understanding and tailoring the parent zeolite prior to copper inclusion are pivotal for finding the maximal methanol yield.

**Figure 1:**
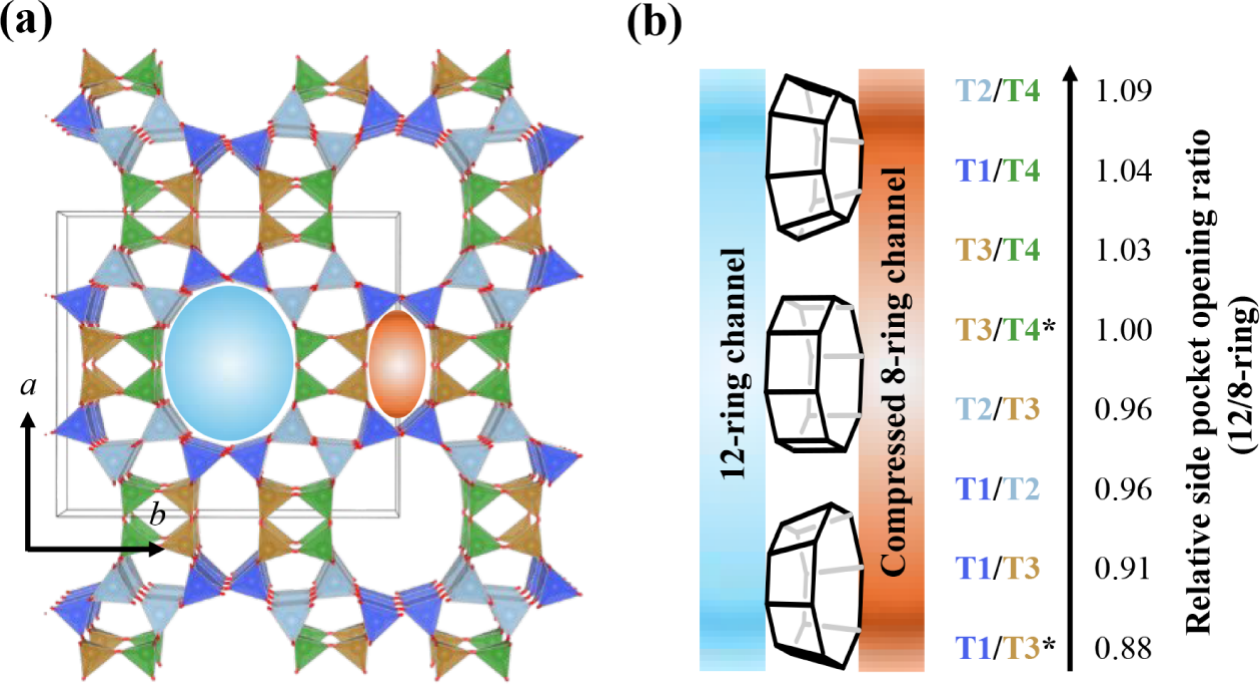
(a) MOR topology showing the four inequivalent T positions labeled as follows: T1: Blue, T2: Gray, T3: Brown, T4: Green. This image was reprinted with permission from Prodinger et al.^47^ Copyright 2022 American Chemical Society. (b) Visualization of the pore geometry trend and each T-pair’s 12-ring/compressed 8-ring side pocket opening ratios.

The reactivity of Cu-MOR systems is highly sensitive to copper speciation. *In situ* X-ray spectroscopy on Cu-MOR during heating to 500 °C in either oxygen or an inert gas suggested the presence of different framework-coordinated Cu­(II) species.^11^ It must also be noted that not all of these species are responsible for the activation of methane.^9^ Fischer et al. exposed the presence of different types of copper species in the MOR structure proposing that the type as well as the reactivity of copper species formed vary as a function of the copper loading in MOR.^17^ Pappas et al. indicated that a dicopper-oxo species, possibly located in the 12-ring channel and the side pocket of the mordenite, is the active species that can oxidize methane in the MTM reaction. Plessers et al. identified three different mono-μ-oxo dicopper [Cu_2_(μ-O)]^2+^ sites: one in the side pocket of the MOR framework exposed to the 12-ring, another in-between the 8-ring side pocket, and the final one embedded in the compressed 8-ring channel. Copper hydroxo species were also reported to be present. They hypothesized that the embedded [Cu_2_(μ-O)]^2+^ site and [CuOH]^+^ are responsible for the activity at lower Cu concentrations.^18^

Mono-μ-oxo dicopper ([Cu_2_(μ-O)]^2+^)^7,19–23^ as well as a copper hydroxo ([CuOH]^+^) pair, have also been suggested based on spectroscopic evidence.^24–30^ Density functional theory (DFT) calculations performed by Wijerathne et al. on Cu-MOR found that with a random Al distribution a variety of copper species are formed, which exhibit different activity toward methane-to-methanol conversion.^31^ In addition to copper speciation, it has also been proposed that the second coordination sphere, similar to enzymes, plays an important role in driving the activity.^23^ These indications of a range of Cu species with varied propensity for MTM have motivated us to develop ways of influencing their speciation within zeolites, specifically mordenite.

In this work, in-house mordenite synthesized with equimolar amounts of sodium and potassium as mineralizing agents (1Na-MOR and 1K-MOR from Prodinger et al., respectively)^15^ was copper exchanged with different copper loadings and tested for activity toward the activation of light alkanes. Advanced spectroscopic techniques, along with reaction tests, were used to investigate the relationship between Al-placement-induced large- and small-port mordenite and their impact on the formation of different active copper sites for methane activation. DFT calculations were conducted on a series of T-site-enriched MOR models to explore the effects of different aluminum positions on the MOR framework geometry, while Monte Carlo simulations were performed to assess whether the copper species incorporated into these materials would vary and corroborate experimental findings.

## Experimental Section

### Material Synthesis and Ion Exchange

Two MOR samples were synthesized, varying the alkali cation type content in the synthesis gel—following the previously reported protocol.^15^ Liquid ion exchange (LIE) of the proton form was then performed by mixing with an aqueous solution of copper acetate (e.g., 0.001 M for Cu/Al = 0.07) at a ratio of 60 mL g_zeolite_^–1^. The samples were stirred for 4 h at room temperature or 60 °C, and the pH was initially adjusted to 5.2 with drops of NH_4_OH. The pH was checked intermittently to ensure it remained close to 5. After completion, the solids were separated via filtration, washed several times with water, and used as is. A list of the samples with different Cu/Al ratios can be seen in Table S3. The nomenclature used for the materials is *x*Cu-MOR­(*y*), where *x* is the Cu/Al ratio and *y* is the parent cation, i.e., Na or K.

### Reaction Tests

All tests were performed using a laboratory-scale plug-flow reactor (inner diameter = 6 mm). Samples were pressed and sieved (4 t, 250–425 μm). 100 mg was loaded into the reactor (dry weight ∼85 mg). The quartz reactor was placed inside a tubular oven, and the temperature was calibrated by placing a thermocouple (K-type) inside the reactor. The reaction protocol was as follows: Oxidation of the copper and dehydration of the zeolite by subjecting the samples to an oxygen flow of 15 mL min^–1^ at 500 °C for 8 h. The sample was then cooled to 200 °C and flushed with an inert gas for 50 min before methane was introduced to the system for 3 h (15 mL/min). Finally, the activated species were extracted by flowing a 10% steam (saturator at 45 °C) in an inert stream (16.5 mL/min in total flow) to form methanol, and 5% benzene vapor (saturator at 6 °C) in an inert mixture (He/10% Ne + 5% C_6_H_6_ (*g*), total flow 15.8 mL/min) for benzene methylation. The effluent from the reactor was monitored with an online quadrupole mass spectrometer (Pfeiffer). For quantification of the products, a Gas Chromatograph (Agilent) was equipped with a PLOT U column, using He as the carrier gas and a flame ionization detector for detection (FID). The performance of the materials tested herein is reported either as the total yield of product produced per gram of zeolite (μmol_*x*_ g_Zeolite_^–1^, eq 1) or as productivity (mol_*x*_ mol_Cu_^–1^, eq 2) of a given stream/extraction time.

1 $${\mathrm{Yield}}_{X}=\frac{\mathrm{\mu mol produced of}X}{g_{\mathrm{Zeolite}}}$$

2 $${\mathrm{Productivity}}_{X}=\frac{{\mathrm{Yield}}_{X}}{{\mathrm{Concentration}}_{Cu}}$$

### X-ray Diffraction

X-ray diffractograms for Rietveld refinements were recorded using a Bruker D8-A25 in transmission capillary geometry with a Ge (111) Johanssen monochromator and Lynxeye detector with Cu K-alpha-1 radiation (λ = 1.5406 Å). The samples were collected at 25 °C after equilibration with ambient moisture, followed by water removal at 350 °C for 3 h inside flame-sealed capillaries. Fourier map analysis was performed with TOPAS v6, and the empty framework model was adapted from the IZA-SC Database.

### Scanning Electron Microscopy

Backscattered electron (BSE) imaging was used to investigate the samples for the Cu nanoparticles. A Hitachi SU8230 microscope was used to obtain the images using an acceleration voltage of 1 kV and a current of 30 μA. On the same instrument, Energy Dispersive X-ray (EDX) mapping was used as a secondary elemental analysis to investigate the distribution of Cu on the samples.

### X-ray Absorption Spectroscopy (XAS) and MCR Analysis

*Operando* Cu K-edge XAS spectra were collected in transmission mode on the Swiss-Norwegian beamline (BM31) of the European Synchrotron Radiation Facility (ESRF) in Grenoble.^32^ The beamline was equipped with an air-bearing liquid nitrogen double-crystal monochromator (two flat Si[111] and a flat Si[311] pairs) and ionization chambers filled with a He/Ar mixture.^33^ The XAS spectra were acquired with ca. 3 min/scan acquisition time, and the collected data were normalized to unity edge jump at the Cu K-edge using the Larch package.^34^ The samples were pressed and sieved to a 160–190 μm fraction and placed inside 1.5 mm capillaries that were glued to a specialized bracket connected to the gas lines via a Swagelok connection. Quartz wool was placed on each side of the sample to secure it. The temperature was controlled by a heat blower installed at the beamline, ensuring homogeneous heating throughout the capillary volume. The qualitative assessment of potential leaks in the gaseous environment was analyzed with an online Mass Spectrometer (MS, Pfeiffer) also provided at the beamline.

The experimental procedure for the first cycle was as follows: samples were exposed to a flow of 5 mL/min of pure O_2_ at room temperature (RT). The temperature was then ramped up to 500 °C, with concurrent XAS spectra collection. The system was then cooled to 200 °C, maintaining the O_2_ flux and continuously monitoring the sample by XAS. A subsequent 50-min He purge eliminated any possible residual O_2_.The reducing agent (CH_4_) was sent to the sample and maintained for 3 h. Next, He purge and finally H_2_O as steam was introduced to extract the resultant products. The second cycle began by reintroducing He at 200 °C and increasing the temperature to 500 °C without oxygen, followed by the subsequent steps as described above.

The experimental energy range (8800–9030 eV) of normalized μ­(*E*) XAS spectra corresponding to the XANES spectral regions was analyzed via the MCR-ALS method. MCR-ALS reconstruction utilized the MATLAB-based MCR-ALS Graphical User Interface (GUI) developed by Jaumot et al., employing MATLAB R2022.

### Electron Paramagnetic Resonance (EPR) Spectroscopy

Cu-MOR samples were activated following the procedure reported by Fischer et al.^17^ About 25 mg of the sample was placed into a cell used for the EPR measurements. The cell was evacuated under vacuum (<10^–4^ mbar) at 500 °C for 4 h to dehydrate the sample, followed by a calcination step in 400 mbar of O_2_ for 4 h. A second dehydration step followed the calcination, lasting 4 h under vacuum. At the end of the treatment, the EPR tube was sealed off.

X-band (microwave frequency 9.45 GHz) CW-EPR spectra were acquired at 77 K on a Bruker EMX spectrometer equipped with an ER 4119 HS cylindrical cavity and a Bruker EMXmicro spectrometer. In both cases, a modulation frequency of 100 kHz, a modulation amplitude of 1 mm, and a microwave power of 2 mW were adopted. The Spin Count package of Bruker Xenon Software was employed to perform the EPR quantification.

Pulse EPR measurements were performed at 10 K at X-band (microwave frequency 9.75 GHz) on a Bruker ELEXSYS 580 spectrometer equipped with a helium gas-flow cryostat from Oxford Inc.

X-band electron spin echo (ESE) detected EPR spectra were acquired using the pulse sequence π/2*–*τ–π–τ*–*echo. Pulse lengths of *t*_π/2_ = 16 ns, *t*_π_ = 32 ns, and a τ value of 200 ns were used in conjunction with a shot repetition time of 3.55 kHz.

*Phase memory times* (*Tm*) were measured by the Hahn Echo sequence by increasing the interpulse delay τ starting from τ = 110 ns. Typical pulse lengths were *t*_π/2_ = 40 ns and *t*_π_ = 80 ns.

*Spin–lattice relaxation times* (*T*_1_) were measured using the standard inversion recovery sequence (π–*t*_d_–π/2–*t*–π–*t–*echo), with π/2 = 16 ns.

X-band Hyperfine Sublevel Correlation (HYSCORE^35^ spectroscopy measurements were carried out with the standard pulse sequence π/2−τ–π–π/2*–*t_1_–π–t_2_ *–*π/2*–*τ*–*echo, employing an eight-step phase cycle to delete unwanted echoes. Pulse lengths *t*_π/2_ = 16 ns, *t*_π_ = 32 ns, and a shot repetition time of 1.77 kHz were used. The increment of the time intervals *t*_1_ and *t*_2_ was 16 ns, starting from 80 to 2704 ns, resulting in a data matrix of 170 × 170. The τ value used for each measurement are reported in the figure captions.

The time traces of HYSCORE spectra were baseline corrected with a third-order polynomial, apodized with a Hamming window, and zero-filled to 2048 points. After the 2D Fourier transformation, the absolute-value spectra were calculated. All the EPR spectra were simulated using the EasySpin^36^ package (version 6.0.0-dev.34) running in MATLAB.

### Diffuse Reflectance Infrared Fourier Transformed Spectroscopy (DRIFTS)

DRIFTS was performed on a Bruker Vertex 70 instrument fitted with a liquid nitrogen-cooled mercury cadmium telluride (MCT) detector. A pressed and sieved sample was mounted in a Harrick high-temperature reaction chamber fitted to the Praying Mantis DRIFTS cell. Samples were first heated in 15 mL min^–1^ O_2_ to 500 °C for 2 h (ramp rate = 10 °C min^–1^) before being cooled to 300 °C, and spectra were collected. A reference sample of KBr with similar sieve fractions was measured at comparable temperatures and atmospheres to obtain the background spectra.

### DFT Simulations of Large-Port and Small-Port Mordenite

Calculations were performed using version 6.1 of the CP2K software package,^37^ which uses Gaussian-type pseudopotentials and the plane waves method. Perdew–Burke–Ernzerhof (PBE) exchange-correlation functional with a Grimme dispersion correction D3 was employed.^38,39^ Plane wave cutoff of 800 Ry and relative cutoff of 60 Ry were set to ensure the convergence of the studied systems.

The structure studied is orthorhombic, with unit cell parameters of *a* = 18.1651 Å, *b* = 20.3785 Å, and *c* = 7.4934 Å. For periodic calculations, a 1 × 1 × 1 cell was used, with Si/Al = 5 and Si/Al = 11, featuring different aluminum distributions. Models of H-MOR were created with aluminum (Al) located in either the T1, T2, T3, or T4 sites and with Brønsted Acid Sites (BAS) on the adjacent oxygen between Al and Si atoms. The aluminum distribution is denoted as “T*x*/T*y*,” indicating which two T sites are occupied by Al, with an equal number of Al atoms in both sites.

Geometry optimizations were performed to determine the minimum energy configuration. Following geometry optimization, additional cell optimization calculations were performed to allow the unit cell to relax, thereby accounting for more realistic changes in the catalyst material. The angles were kept fixed for all cells during these optimizations.

To examine the distortion of the framework influenced by varying Al site locations, we measured the ratio of the longest and shortest distances between opposite oxygen atoms of the 12-ring channel and 8-ring channel. Additionally, we measured the area of the pore opening toward 12- and 8-ring channels (also referred to as the “side pocket”) using the following equation, where *A* represents the area of the side pocket, and *r̅* is the average oxygen–oxygen (O–O) distance of each opening.

$$A=\pi \frac{{\mathrm{r̅}}^{2}}{4}$$

The O–O distances were measured from the oppositely positioned oxygen atoms and by subtracting each O atom’s Van der Waals radius (2 × 1.35 Å). Finally, we evaluated the ratio of the area of the 12-ring channel side pocket to the area of the 8-ring channel side pocket. This quantitative assessment allows us to establish structural changes between all studied cases of the H-MOR with varying Al distribution, associated with either large-port or small-port mordenite.

### Monte Carlo Simulations of Copper Species

To estimate the Cu cation speciation under thermodynamic equilibrium for different Al distributions (MOR­(K) and MOR­(Na)), we used Monte Carlo simulations with Cu species formation probabilities calculated at 773 K using the method described in our previous work.^31^ In the simulations, the Al distributions were randomly generated while maintaining specific ratios of Al in different T-sites (Table S4) for all the Si/Al ratios explored. Cu was exchanged as a dimer ([Cu_2_O]^2+^, [Cu_2_O_2_]^2+^, [Cu_2_O_2_H_2_]^2+^, [Cu_2_OH]^2+^) or a monomer ([Cu]^2+^ or [CuOH]^+^) at each 2Al configuration until all of the Al sites in the zeolite lattice were occupied. The presence of “isolated Al” is explicitly included in the simulations because 2Al configurations that are farther than 10 Å apart are allowed to exchange only [CuOH]^+^. Subsequently, the percentage Cu exchanged as each dimer (or monomer) and the total percentage of dimers at any Cu/Al were calculated by averaging 5000 independent Monte Carlo simulations.

### C–H Activation Energy at Different Copper Dimers

To explore possible correlations with experimental MeOH yields at different Cu loadings, we calculated the dimer Cu–O–Cu angles and C–H activation barriers. The Cu–O–Cu angles of the populated dimer motifs corresponding to each sample were calculated using their corresponding DFT-optimized dimer structures. The C–H activation barriers were determined for each Cu dimer site using the DeePMD^40,41^ derived machine learning potential that was specifically trained for C–H activation of CH_4_ in Cu-exchanged zeolites.^42^ To make the initial guess of adsorbed, activated, and dissociated CH_4_, we placed CH_4_ near Cu–O–Cu dimers and randomly rotated CH_4_ while maintaining previously reported C–H and Cu–O–H distances. Initial guesses with distances between framework atoms and adsorbed CH_4_ less than 1.0 Å were rejected, as they could lead to undesirable higher energy structures. We explored 100 total random guesses (details in Section S9) for each 2Al configuration to position the CH_4_ near the Cu–O–Cu dimer and minimized the potential energy (evaluated by DeePMD force field) using SciPy’s basin hopping algorithm^43,44^ with a parameter temperature (*T*) of 10 and a step size of 0.3 Å to perturb distances, followed by local optimization with “L-BFGS-B” optimizer in 1000 basin hopping steps. We then selected the minimum energy initial state and its corresponding transition and final states for Nudge Elastic Band (NEB) calculations (Figure S11). All the initial guesses for adsorbed and dissociated CH_4_ were optimized with the pretrained DeePMD force field to forces less than 0.03 eV/Å, and the NEB calculations were performed using the Atomic Simulation Environment’s^45^ FIRE algorithm with forces less than 0.03 eV/Å to locate the transition state. All the structure files and a sample code for activation energy calculation are provided in the Supporting Information.

## Results

### Geometry Optimization as a Function of Al Siting

As previously suggested by X-ray diffraction Rietveld refinement,^15^ MOR­(Na) has T1 and T3 sites preferentially populated by Al. This material also exhibits large-port behavior (a toluene uptake >4.5 wt %). In contrast, MOR­(K) does not favor incorporation at the T1 and T3 sites, instead being biased toward the T2 and T4 sites, incidentally reflecting a more random Al distribution (all sites have a nearly equal chance of being populated). Figure 1 shows the inequivalent T positions at which Al could be situated. To investigate the impact of these distinct Al positions on the local geometry of MOR, a DFT study was conducted on idealized cases of H-MOR, where Al is placed in specific T-site position pairs (e.g., T1/T3) at an Si/Al ratio of 5 and 11. Following the calculation methodology described in the Experimental Section, we carried out the geometry and cell optimization algorithms.

For the Si/Al = 5 ratio, several distinct pairs were examined: T1/T3, T2/T3, T3/T4, T1/T2, T2/T4, and T1/T4, as well as two alternate configurations of T1/T3 and T3/T4 (T1/T3* and T3/T4*, respectively). To assess pore structure variations, the long and short axes of the 12-ring and compressed 8-ring channels were measured, along with the opening area of the side pocket toward each channel. These were calculated based on the average O–O distance of each opening, measured between oppositely positioned oxygen atoms, with van der Waals radii subtracted (2 × 1.35 Å).^14,46^ The distances used to determine the 12-ring and compressed 8-ring pore dimensions are visualized in Figure S1, and the resulting pore dimensions are summarized in Table S1. It is important to note that the oxygens in the MOR topology are not aligned in the same cross-sectional plane. Consequently, the reported distances are not exactly perpendicular to the straight channels and should be regarded as approximate.

The “large-port” T1/T3-enriched mordenite features a relatively compressed 8-ring and an oval 12-ring. In contrast, the “small-port” T2/T4-enriched structure has a more open 8-ring and a larger 12-ring. The most notable geometric variation is observed in the side pocket shape, as illustrated in Figure 1b. The side pocket of the T2/T4-enriched framework adopts a conical shape, with the smaller opening oriented toward the compressed 8-ring (12-ring/8-ring opening ratio: 1.09). Conversely, the T1/T3-enriched side pocket is also conical but with reversed ratios (12-ring/8-ring opening ratio: 0.91). In comparison, the side pocket is cylindrical for T3/T4 (and T3/T4*) with ratios of 1.00 and 1.02, respectively. Incidentally, the small-port (T2/T4) configuration exhibits a larger unit cell volume (2873 Å^3^) than the large-port variant (2782 Å^3^).

As the Si/Al ratio is increased to 11, the geometric distortions diminish due to the reduced number of Al atoms in the framework (Table S2). However, this trend persists. Based on the pairs investigated (see Tables S1 and S2), it becomes clear that T4 and T3 are the deciding sites for the shape of the side pocket for both Si/Al ratios; Al in the T4 position makes the side pocket a conical shape with a bigger opening toward the 12-ring channel, Al at position T3 has the opposite effect. As seen by Prodinger et al.,^15^ the port size has an influence on the catalytic activity of the H-form of the materials toward isobutane cracking, with the large-port MOR exhibiting higher initial conversion than the small-port MOR suggesting more acid sites in the 8-ring where partial confinement favors conversion. This suggests that the differences in port size, also observed with DFT calculations, can hypothetically have a second-sphere effect on the copper species present during the methane-to-methanol reaction. The rest of this contribution will now attempt to delineate the effect of this geometric distortion on the incorporation, speciation, and reactivity of Cu in MOR zeolite with respect to methane activation.

### Copper Incorporation

The active Cu-MOR material is prepared via liquid ion exchange (LIE) on the H-form of the starting materials, ensuring no cation—in this case K or Na—is present in the MOR before copper incorporation and further testing, with the exchange isotherms shown in Figure S3. Prodinger et al.^15^ reported that the M^+^/Al^3+^ ratio prior to copper incorporation is well below one, with temperature-programmed desorption profiles of propylamine exhibiting comparable acidity, thus confirming the absence of cations in the subsequent materials that are copper-incorporated and tested for the MTM conversion in the MOR samples. MOR­(Na) exhibits a higher copper uptake at both room temperature and 60 °C, following a Langmuir-type isotherm. In contrast, MOR­(K) displays behavior more akin to a Henry’s isotherm at 25 °C. However, as the temperature is increased to 60 °C, Cu incorporation rises, and MOR­(K) begins to show a Langmuir-type isotherm, up to a copper solution excess concentration (i.e., Cu^2+^/[H^+^]_2_ Al ratio) of about 500. These results suggest a stronger adsorption constant in the case of the MOR­(Na) material, which could be related to both the strength of sites and/or diffusion limitations.

Previous analysis of the crystal morphology of these materials via scanning electron microscopy showed differences in the aspect ratio, c/a, of the crystallites.^15^ While the overall crystal size was comparable for both samples (ca. 2000 μm^3^), the presence of K^+^ was suggested to accelerate the growth rate of the *c*-axis forming cubic crystals, leading to a ≈ c with small-port (<4.5 wt % toluene uptake) behavior, while Na^+^ ions resulted in more plate-like crystals with c < a and large-port behavior. Ion exchange is governed by Coulombic interactions, where strong interactions result in higher adsorption constants and increased uptake at low ion concentrations, as seen for MOR­(Na). Due to the large size of the hydrated Cu^2+^ ion ([Cu­(H_2_O)_6_]^2+^), its diffusion through the small-port mordenite pores is restricted, reducing the degree of ion exchange and leading to a Henry-like uptake behavior. At elevated temperatures, these diffusion barriers can be overcome, allowing ion exchange to be driven by strong Coulombic interactions between the Cu ion and acidic exchange sites. Thus, we posit that the varying extents of Cu exchange can be attributed to intrinsic material differences originating from the aluminum distribution. Successful copper incorporation is confirmed by performing elemental analysis, and we also verified that no Cu metal nanoparticles were present using backscattered electrons with EDX. After LIE, the copper incorporation was further validated with diffuse reflectance infrared Fourier transform spectroscopy (DRIFTS) (Figure S4). A splitting of the peak at around 1000 cm^–1^ can be seen in both mordenites after Cu is introduced. For CHA, vibrations at 900 and 950 cm^–1^ have been attributed to asymmetric T–O–T vibrations perturbed by Cu­[II] from Cu^2+^ and [CuOH]^+^, respectively.^48–51^ While similar signatures have not been explicitly reported for the mordenite framework, EPR results (vide infra) agree with these assignments for the mordenite framework. Backscattered electron imaging (BSE) and energy-dispersive X-ray (EDX) mapping were used to examine the Cu distribution in the samples. No evidence of nanoparticle formation was observed in the BSE images across the different materials. Although EDX maps showed brighter regions at higher Cu loadings, these do not definitively indicate a gradient in Cu distribution (Figure S12).

### Methane-to-Methanol Activity

The Cu-MOR activated at 500 °C in O_2_ and CH_4_ exposed at 200 °C exhibits a volcano-shaped curve, consistent with previous Cu-MOR.^47^ Notably, the MOR­(K) sample reaches a maximum at ca. 0.07 Cu/Al (140 μmol_Cu_ g^–1^_zeolite_), while the MOR­(Na) sample reaches a maximum at 0.125 Cu/Al (265 μmol_Cu_ g^–1^_zeolite_). This shift suggests that a greater proportion of inactive copper species is incorporated in the MOR­(Na) sample. As per Table S3, we see that the trend of e produced methanol increases with increasing copper loading. However, when we take into consideration the amount of methanol vs the copper loaded—thus the productivity—we see that increasing the copper loading beyond the maximum, as shown below, leads to more methanol but not in proportion to the amount of copper, meaning that more inactive species are added beyond this point. When comparing MOR samples with similar copper contents highlighted by the circles in Figure 2, the MOR­(Na) shows a lower productivity of 0.24 mol_methanol_ mol_Cu_^–1^, compared to the MOR­(K), which has a productivity of 0.48 mol_methanol_ mol_Cu_^–1^. It is important to note that all subsequent studies and comparisons exclusively focus on these two samples (both with Cu/Al ratio of 0.07, highlighted in the plot) in order to isolate the effect of structural factors of the materials independent of the copper loading.

**Figure 2:**
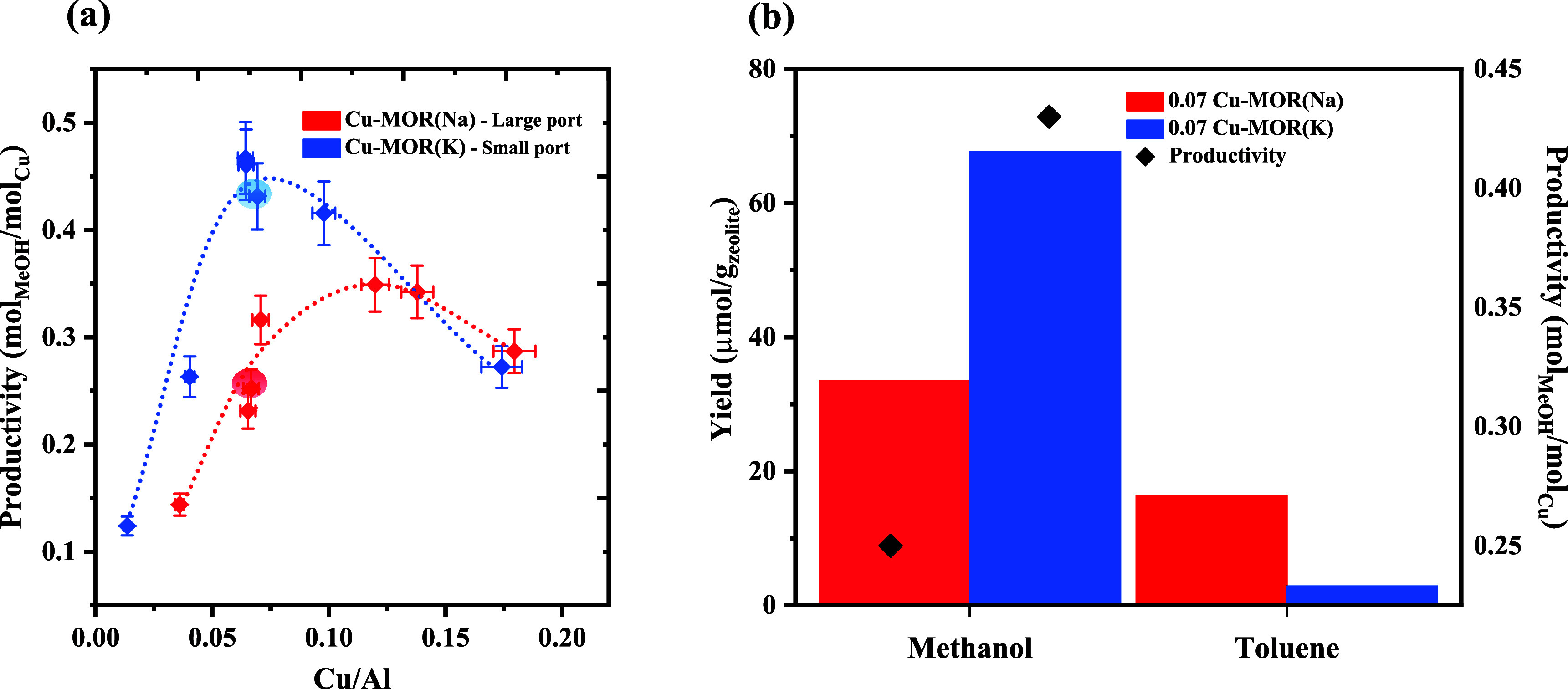
(a) Methanol productivity as a function of the copper content. Error margins are obtained by repeated tests on the same sample. (b) Bar graph illustrating the yield of methanol when using water (to extract the methoxy) and the yield of toluene when using benzene for the materials containing the same Cu/Al ratio of 0.07. Corresponding methanol productivities (black diamonds) are also indicated.

To probe the location and accessibility of active sites, benzene was employed as a bulkier extraction molecule, with a kinetic diameter of 0.58 nm, compared to 0.265 nm for water. Due to its larger kinetic diameter compared to that of water, benzene enables selective probing of more spatially confined sites within the material.^52^ Extraction with benzene led to the formation of toluene, enabling a direct comparison of toluene and methanol yields when methoxy species were extracted using benzene and water, respectively. As shown in Figure 2b, both samples (Cu/Al = 0.07) lose activity, albeit the loss is significantly larger for the small-port MOR­(K). Only 49% and 4% of methoxy react with benzene compared to steam for MOR­(Na) and MOR­(K), respectively (16 μmol_Toluene_ g^–1^_zeolite_ and 3 μmol_Toluene_ g^–1^_zeolite_ for the two samples).

Note that the port size is characterized by how much benzene or toluene the MOR framework can adsorb. We previously determined this value experimentally using toluene vapors and finding it to be 250 μmol/g toluene for the small-port and 550 μmol/g toluene for the large-port variants.^15^ Despite these known differences, the substantially lower toluene yield from Cu-MOR­(K) cannot be fully explained by adsorption limitations alone. The disparity suggests that steric hindrance within the more confined pore of the small-port variant restricts the formation of the bulkier toluene molecule. These results indicate that the methoxy species, and consequently the active sites, are situated in more sterically hindered, constrained regions in Cu-MOR­(K) compared to Cu-MOR­(Na).

### Determining the Reactivity of the Copper Species

To further investigate the reactivity of the Cu species, we performed *operando* X-ray absorption spectroscopy (XAS) to follow the Cu K-edge, with each sample undergoing two reaction cycles (Scheme S1). In the first reaction cycle, the samples were activated in O_2_, before methane activation and extraction, and in the second cycle, under inert He, both at 500 °C with a heating rate of 10 °C/min. These conditions were used to evaluate the samples’ reducibility and their ability to reoxidize using a soft oxidant H_2_O (left in the pores after extraction of products). As illustrated in Figure 3a–b, both samples predominantly exhibit a Cu­(II) state as they are heated in O_2_, evidenced by the pre-edge 1s → 3d transition peak located at 8977 eV.

**Figure 3:**
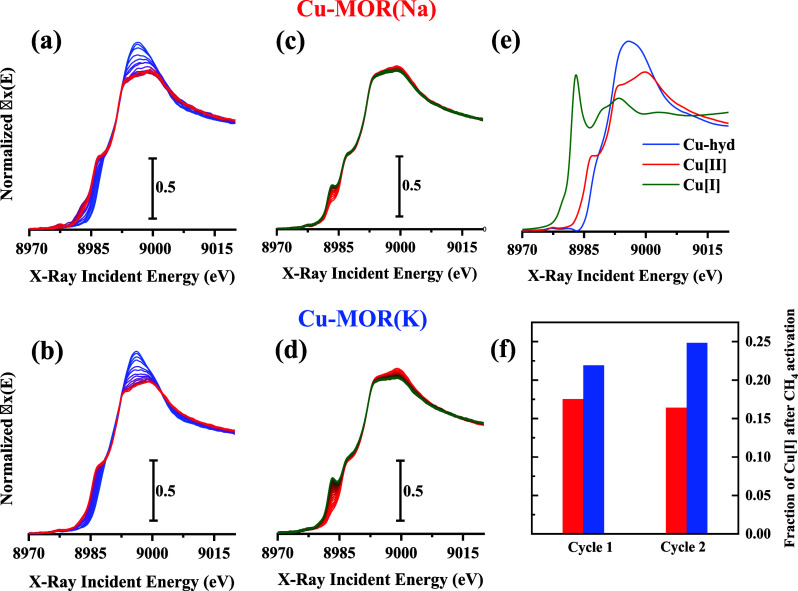
XANES spectra of Cu-MOR (Na) (a, c) and Cu-MOR (K) (b, d) during oxygen activation (a and b) and CH_4_ activation (c and d). The pure spectra obtained for MCR analysis are depicted in (e), and the fraction of Cu­[I] obtained from MCR analysis for both samples (blue: Cu-MOR­(K) and red: Cu-MOR­(Na)) during the first cycle (oxygen activation) and second cycle (inert activation) in (f). Spectra in (a) and (b) transition from blue (Cu-hyd) to red (Cu­[II]), while (c) and (d) transition from red (Cu­[II]) to green (Cu­[I]).

Upon oxygen activation, the 1s → 4p transition peak at 8985 eV, associated with Cu­(II) ions, increases, accompanied by a reduction in the white line (WL) intensity. These changes suggest structural modifications in Cu­(II) species, likely due to the dissociation of coordinated ligands in the primary coordination shell, indicating new Cu­(II) speciation. After O_2_ activation, a small peak at 8982 eV appears, characteristic of the 1s → 4p transition of Cu­(I), consistent with the autoreduction of Cu­(II) during cooling from 500 to 200 °C.^53–55^

Regarding methane activation reported in Figure 3c–d, an increase in the Cu­(I) species concentration is observed, with intensity differences between the samples. Multivariate Curve Resolution (MCR)^56–58^ identified three key components: hydrated ([Cu­(H_2_O)_6_]^2+^), (Cu-hyd), framework-coordinated Cu­(II), and framework-coordinated Cu­(I) (hereafter referred to as Cu­(II) and Cu­(I), respectively). The concentration profiles of these species during the experiment are shown in Figure S5a. Methane exposure led to Cu­(I) formation in both samples, although the rate of formation differed. The Cu-MOR­(Na) sample reduces to Cu­(I) readily, reaching its final fraction of Cu­(I) almost immediately, whereas Cu-MOR­(K) reduces more slowly (Figure 7). As demonstrated by Bregante et al.^59^ and Lomachenko et al.,^60^ there is a direct correlation between oxygen activation time and methanol yield. Accordingly, the shorter oxygen activation period used during the in situ XAS measurements (2 h) results in low Cu­(I) formation, as seen in Figure S5a. This observation is consistent with the findings reported by Lomanchenko et al.^60^ The increased prevalence of the Cu-hyd species in the Cu-MOR­(K) sample, as shown in Figure S5a, is attributed to water contamination during the in situ XAS measurements, consistent with the MS data presented in Figure S5b.

After 3 h in the methane feed, the Cu-MOR (K) exhibited a higher fraction of Cu­(I) aligning with the increased activity observed during methane reaction tests (see above, Figure 2). When water was introduced to the sample, Cu-hyd dominated both samples as methanol is formed from the methoxy species reacting with water, although some unreacted Cu­(II) persisted (Figure S5a). Upon reactivating the samples in an inert state for the second reaction cycle, Cu­(II) reappears in both samples, though not to the same extent as before, leaving some copper as Cu­(I) as shown in Figure S5a. According to Sushkevich et al., the residual water left in the pores after extraction has been reported to facilitate reoxidation of the sample; however, in this case, complete reoxidation was not achieved.^61^

In summary, XAS findings demonstrate that Cu-MOR­(Na) and Cu-MOR­(K) differ in their relative extent of Cu­(I) formation after 3 h of methane exposure, which nicely correlated with the productivity differences shown in Figure 2. This suggests that altering synthesis conditions to introduce an Al bias results in copper species with varying reducibility and potential for activating methane, requiring deeper understanding. The rate of Cu­(I) formation also varies between the two samples and is likely attributed to differences in the local confinement environments of the copper species, as suggested by the toluene experiments and the copper exchange differences across the samples. Capillary XRD was collected on these 2 selected samples upon dehydration at 350 °C and flame sealing. Difference Fourier map analysis shows electron density clouds inside the side pockets, and the two samples appear very similar, perhaps reflecting that the dominant copper species are, on average, comparable (Figure S6). No Rietveld refinement model is being proposed; note that we believe multiple copper–oxygen species and local configurations are enclosed within the side pocket, and therefore those electron density clouds do not represent specific atomic positions. The findings here align with those from XAS highlighting the similarity of the copper species on average. This suggests that the activity differences, observed experimentally, should be associated with subtle differences in pore geometry or species geometry, which are not captured by these averaging, bulk techniques.

### Probing the Copper Species Geometry

Due to the low copper content of the samples, no information could be gained from the EXAFS region in the XAS spectrum, precluding any information on the geometry of the Cu atom from being obtained. Instead, we turned to EPR spectroscopy, a technique that is sensitive to the geometry, speciation, and siting of isolated copper in +2 oxidation state. This makes it possible to study and visualize differences brought about by the different cations used during synthesis. The caveat is that aggregated paramagnetic Cu­(II) leads to unresolved broadened lines, while antiferromagnetically coupled Cu–O–Cu pairs result in EPR-silent species^62–66^ allowing for the selective study of monomeric Cu­(II) (e.g., [Cu]^2+^ or [CuOH]^+^) species. It is important to note that monomeric copper species here refers to copper ions that exist in a singular, nonclustered form within a given environment or material.

The results (Figure 4) show the relevant portion of the EPR spectra (*g*_||_/*A*_||_ region) with spin-Hamiltonian parameters similar to those reported for Cu-MOR by Fischer et al.^17^ The two samples studied do not show significantly different EPR features, with the spectra being almost identical and dominated by two species previously assigned by Fischer et al. to monomeric, extra-framework [Cu]^2+^ and [CuOH]^+^ labeled S1 g_||_ 2.32 and S3 g_||_ 2.27, respectively. Interestingly, the Cu-MOR­(K) sample does have a larger concentration of these EPR-visible species. Incidentally, we also probed the samples using DRIFTS as already mentioned above (see Section S5 and Figure S4), finding a splitting of the peak at around 1000 cm^–1^ in both samples. This has previously been suggested, for Cu-CHA, to be due to the presence of [Cu]^2+^ and [CuOH]^+^ species, and while there have been no assignments performed specifically for MOR, the similarity in spectra features aligns well with the observations made from EPR.

**Figure 4:**
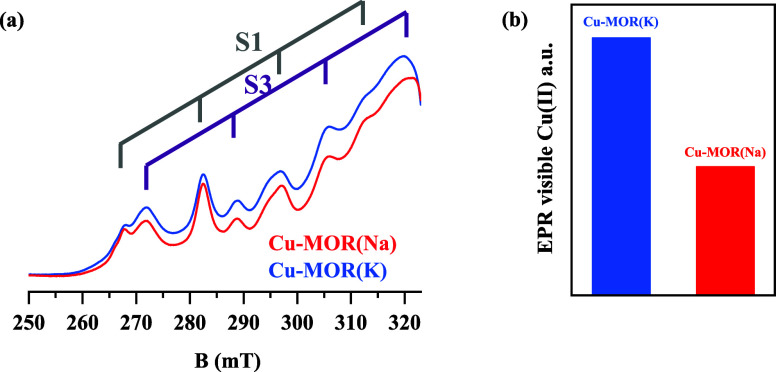
CW EPR spectra of Cu-MOR­(K) and Cu-MOR­(Na) after oxygen activation are shown in (a). The peaks S1 with g_||_ 2.32 and S3 with g_||_ 2.27 from Fischer et al. are shown in purple and red.^30^ The two spectra are normalized to the prepeak at 250 mT. The amount of EPR-visible species is quantified as shown on the right-hand side (b).

To better understand the Cu­[II] species, Electron Spin Echo (ESE)-detected EPR experiments were carried out (see Section S8). The ESE-EPR spectrum allows filtering out fast-relaxing components, associated in this case with dipole–dipole interactions between nearby Cu­(II) ions. Increasing the distance between the copper species will also lead to increased relaxation times. A comparison between the CW-EPR and the ESE-EPR spectra (Figure S7) indicates that a significant fraction of fast-relaxing Cu­(II) is present for both samples, while also exhibiting subtle differences between them. The electron spin relaxation times *T*_1_ and *T*_m_ for paramagnetic Cu­(II) (Figure S8) are always longer for Cu-MOR­(Na), which indicates that the monomeric Cu­(II) species are more spatially isolated compared to the Cu-MOR­(K) sample. We assign this observation to the presence of more bare [Cu]^2+^ species in Cu-MOR­(Na), while the Cu-MOR­(K) sample, by contrast, has more [CuOH]^+^ species. Note that bare [Cu]^2+^ species have been shown to be inactive for activating methane in the case of Cu-CHA, thus pointing toward the activity differences we observe here experimentally.^53^

To assess the local environment of monomeric Cu­(II), Hyperfine Sublevel Correlation Spectroscopy (HYSCORE) was also performed (Figure S9), which allows the detection of small hyperfine interactions from magnetic nuclei (e.g., ^1^H, ^27^Al) surrounding the paramagnetic monomeric Cu­(II) species.^64,65^ For both samples, interactions with nearby ^27^Al and ^1^H nuclei were detected. Importantly, the ^1^H hyperfine interactions observed are consistent with those observed for Cu-CHA^65^ and are diagnostic of monomeric hydroxo-Cu­(II) species. This confirms the assignment previously postulated by Fischer et al., based on the similarity to the features in Cu-CHA.^17^

It is important to note that the vacuum treatment at high temperature for EPR analysis can lead to partial autoreduction of Cu species. However, this effect can be considered minimal in our case for two reasons: according to a study by Sushkevich et al.,^67^ samples with our specific copper loading and Si/Al ratio would experience very low autoreduction at 500 °C, as well as the fact that both samples in our study have the same copper loading and underwent identical treatment conditions. Given these factors, we can reasonably assume that any differences observed between the samples are not significantly influenced by autoreduction effects, allowing us to focus on other aspects of their behavior and properties.

Summarizing, the EPR study indicates that in both samples, monomeric Cu­(II) is incorporated in the form of both extra-framework [Cu]^2+^ cations and [CuOH]^+^ species in proximity to Al^3+^ cations. The analysis demonstrates the potential impact of the pore geometry (small-port and large-port) as observed from the differences in relaxation. Notably, the relaxation times indicate that the MOR­(Na) contains a larger proportion of isolated, inactive [Cu]^2+^ species, while MOR­(K) has a higher concentration of [CuOH]^+^ species, which are active in the MTM reaction.^48^

To complement the experimental insights on Cu speciation from EPR, we then used Monte Carlo simulations (details in the Experimental Section) to explore the possibility of Cu speciation differences (considering different types of Cu monomers and dimers) related to the underlying Al bias introduced during synthesis. In line with the DFT geometry optimizations, we also used the idealized framework structures for MC. We limited the Al incorporation to the T1/T3 and T2/T4 positions to simulate the idealized MOR­(Na) and MOR­(K), respectively. Figure 5 shows that Cu-oxo dimers, [Cu_2_(μ-O)]^2+^, are the majority species in both samples, consistent with their similar XAS spectra. However, our results suggest that Cu-MOR­(K) has a higher fraction of Cu dimers than Cu-MOR­(Na), which forms more [Cu]^2+^, an inactive monomer species for methane activation,^12,53,68^ in line with our interpretation of the EPR data. These findings then allow us to rationalize the lower experimental MeOH productivity of Cu-MOR­(Na) by connecting it to the higher concentration of bare [Cu]^2+^, while attributing the higher MeOH productivity observed for Cu-MOR­(K) (Figure 2a) to its higher [Cu_2_(μ-O)]^2+^ dimer population (following 500 °C oxygen activation) than Cu-MOR­(Na) at a given Cu/Al.

**Figure 5:**
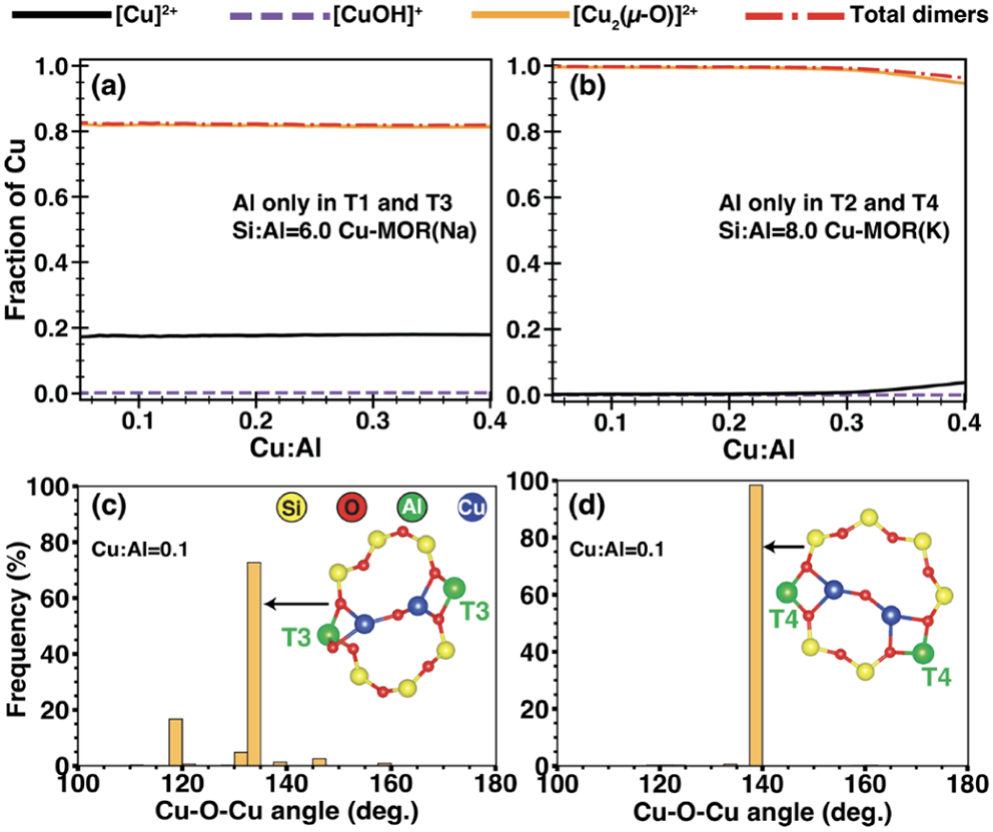
Thermodynamic predictions of Cu cation speciation in MOR zeolites at *T* = 773 K, *P*_H2O_ = 10^–8^ kPa, and *P*_O2_ = 20 kPa for Al distributions consistent with (a) MOR­(Na) and (b) MOR­(K). The Cu–O–Cu angle distribution at Cu/Al 0.1 (approximately 250 μmol/g_zeolite_) for each sample is shown in parts (c) and (d), respectively.

Despite this, the total Cu dimers in both idealized Al-biased configurations are insensitive to the Cu loading within a Cu/Al range of 0.01 to 0.3, consistent with the observation of several groups that dimers are the majority species across a large composition range.^21,58,69,70^ Therefore, as with prior comparisons,^31^ the experimentally observed volcano-type variation of mol MeOH/mol Cu vs Cu/Al cannot be explained solely based on Cu dimer concentration. However, the Cu–O–Cu angles simulated for the MOR­(K) and MOR­(Na) samples indicate that the dimer structures in the two samples are structurally different (Figure 5c–d). These variations in Cu–O–Cu angles may lead to different C–H activation barriers,^42,71,72^ potentially resulting in different MeOH yields at the same Cu/Al.

To test this hypothesis, we computed the activation energies of dimers identified in our Monte Carlo simulations. The activation energies for C–H activation, weighted by their thermodynamically predicted populations, varied from 40 to 160 kJ mol^–1^ in both samples (Figure 6). The activation energy distribution shows a lower average C–H activation barrier (<*E*_a_≥ 67 kJmol^–1^) for Cu-MOR­(K) than for Cu-MOR­(Na) (<*E*_a_≥ 76 kJmol^–1^) for the Cu/Al ratio of 0.02 to 0.2 (Figure 6a and b, respectively), suggesting that MOR­(K) sample could be more active, as observed in the experiments. However, the activation energy distribution for both K and Na samples does not follow a volcano-type variation, as seen in the MeOH productivity (Figure 2a) when Cu loading is increased, failing to rationalize the experimental observation.

**Figure 6:**
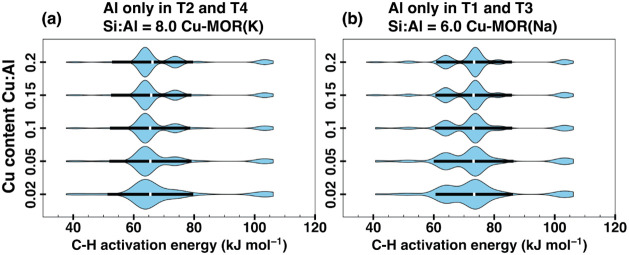
C–H activation barriers of thermodynamically predicted Cu dimers in MOR zeolites at *T* = 773 K, *P*_H2O_ = 10^–8^ kPa, and *P*_O2_ = 20 kPa for Al distributions consistent with (a) Cu-MOR­(K) and (b) Cu-MOR­(Na) at Cu/Al 0.1 (approximately 250 μmol/g_zeolite_). The mean and standard deviation of the distribution are shown as a white square and black thick line, respectively.

The discrepancy between simulations and experiments could be due to some underlying assumptions in our simulations. Notably, previous studies have also reported that the correlation between total Cu dimers and MeOH yield per Cu is weak,^31,73^ which is consistent with our results from MC simulations. For example, despite having low C–H activation barriers, some dimers may not be selective toward MeOH, instead forming other species such as CO_2_.^21,47,74^ Indeed, our previous work suggests that at lower Cu/Al, the selectivity drops to ∼70%, whereas for Cu/Al >0.1 ^47^, selectivity toward MeOH remains high (ca. 90%), which is not captured by the C–H activation barriers alone. Therefore, the lower selectivity toward MeOH formation for samples with Cu/Al <0.1 could be the reason for decreased MeOH productivity at low Cu/Al ratios, as observed in the left-hand side of the peak of the volcano. However, at higher Cu loadings, the selectivity toward MeOH remains high (ca. 90%), and the formation of inactive species could be the reason for decreased MeOH productivity.

Our model also considers all Al to be distributed homogeneously throughout the crystallite and to be accessible for Cu exchange. However, spatial gradients in Cu could hinder dimer and [Cu]^2+^ formation due to the absence of accessible paired 2Al and instead form [CuOH]^+^, which exhibits a different reactivity toward methane oxidation and/or inactive Cu_x_O_y_ clusters in accessible 1Al locations.^12,20^ Nevertheless, our experiments suggest that there is an optimum Cu loading for maximum methanol yield for Cu-MOR despite the total dimer population being relatively constant at low (Cu/Al < 0.3) Cu loadings, consistent with previous observations for Y-zeolites.^75^ Taken together, thermodynamic predictions of Cu dimers cannot explain why there is a volcano-type behavior in MeOH productivity but can rationalize the activity differences between the Cu-MOR­(K) and Cu-MOR­(Na) samples, with the latter having a higher concentration of bare inactive [Cu]^2+^.

## Discussion

We observed pronounced activity differences at low copper loadings in MOR zeolites generated by altering the Al bias. Copper ion exchange is controlled by Coulombic interactions (vide supra), which means that the strongest exchange sites are substituted first. A sample containing a significant number of strong adsorption sites will exhibit a Langmuir-type isotherm, as seen for the Na-MOR sample. Fischer et al. have shown in their EPR study that at very low Cu concentrations, the S1 species dominates with S3 only being populated at higher Cu loadings. S1 essentially is a bare (Cu)^2+^ ion balanced by a nearby Al pair. Incidentally, these kinds of sites are stabilized exceptionally well in the double six rings of the CHA structure, where they have been shown to be inactive in the methane-to-methanol transformation.^53^ The activity data for the Cu-MOR (Na) (Figure 2) shown in this contribution clearly suggest the presence of inactive Cu species at very low copper loadings (up to 60 μmol/g) before they start contributing to the formation of methanol. In contrast, this zone of inactive Cu species is less pronounced in the Cu-MOR­(K) case.

Taking these findings, we posit that the bare Cu^2+^ species are largely inactive, even in the case of MOR. On the other hand, the equally monomeric [CuOH]^+^ species, visible in the EPR spectrum, have been proposed to be more reactive toward methane,^76^ albeit reacting more slowly than mono-μ-oxo dicopper species. Due to the geometry of the side pocket induced by the Al bias, the copper exchange is seemingly affected, with copper incorporation being very facile in the case of MOR-Na but initially leading to a large number of inactive, bare [Cu]^2+^. MOR-K, on the other hand, experiences a more hindered copper uptake, with pore constraints limiting the formation of bare [Cu]^2+^ and instead favoring [CuOH]^+^. This can also be seen from the ESE-EPR studies, where the lesser constrained space in case of the Cu-MOR­(Na) results in longer relaxation times as the copper species are more isolated. The more constrained environment has also been observed when comparing K-MOR and Na-MOR in isobutene cracking as well as propene coking from propylamine-TPD.^15^ Notably, the previously benchmarked Cu-MOR system based on a commercial sample only exhibited its maximum at higher copper concentrations (ca. 250 μmol/g), while also being readily ion exchanged, in line with the observations made here for the MOR-Na system.

It has been shown by Sushkevich et al. that the [CuOH]^+^ species react more slowly, and we also observe this in the slower Cu­(I) formation rate during the operando XAS experiments for the 0.07 Cu-MOR­(K) sample, which contains a higher fraction of [CuOH]^+^ species. Although the XAS measurements were affected by water contamination more significantly in the 0.07 Cu-MOR­(K) sample (Figure S5b), clear differences in both the rate and extent of Cu­(I) formation are still evident. Under fully dehydrated conditions, an even larger disparity in the rate and total number of Cu­(I) formations is anticipated, with the total amount formed expected to better correlate with catalytic performance data obtained under dry conditions. Indeed, we were able to further strengthen this claim by performing the methane-to-methanol reaction under varied methane exposure times. Figure 7 contrasts the fraction of Cu­(I) measured via XAS with the amount of methanol produced as a function of the methane exposure time. The similarity between the trends is self-evident. 0.07 Cu-MOR­(Na) sample achieves its maximum MeOH productivity after 2–3 h in both the reaction test and the operando experiment, whereas 0.07 Cu-MOR­(K) keeps increasing in MeOH productivity, even seemingly coming very close to the 0.5 threshold believed to hold for dimeric Cu-oxo species. As shown in Figure 7b, both materials reach maximum productivity after 3 h of methane exposure. Therefore, this time point was used as a standard reaction duration to ensure full utilization of active sites for methane activation and subsequent methanol production.

**Figure 7:**
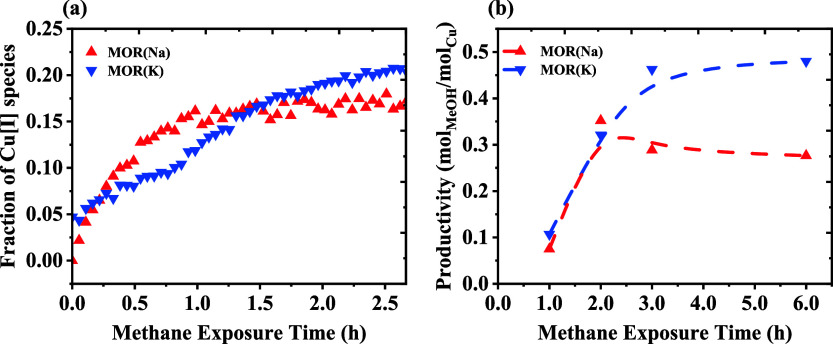
(a) MCR from XAS shows different rates of formation of Cu­(I) and (b) productivity increases when methane exposure time is extended.

The EPR results demonstrate that at the same copper loading, the Cu-MOR­(K) sample has a lower concentration of bare [Cu]^2+^ and thus forms more [CuOH]^+^ species. While bare [Cu]^2+^ is formed at low Cu loadings, the population of dimeric Cu species (e.g., [Cu_2_(μ-O)]^2+^) typically becomes more prominent at higher Cu loadings. MC simulations posit that for the idealized Al-biased configurations, MOR­(K) forms a more reactive dimer structure based on the Cu–O–Cu angle and the activation energies calculated (Figures 5 and 6). Yet, it must be highlighted that not all dimers are active, suggesting the presence of additional species, such as [CuOH]^+^ contributing to the overall activity.^77,78^ Furthermore, our thermodynamic Cu speciation predictions assume that all of the 2Al configurations are accessible for Cu^2+^. However, kinetically formed spatial gradients of Cu species^79^ may hinder 2Al sites located inside the MOR crystallite from fully exchanging Cu, thereby forming other species, such as [CuOH]^+^ in greater amounts than thermodynamically predicted. While such gradients were not observed in the EDX analysis, potentially due to the limited sensitivity of the technique in detecting subtle variations in Cu distribution within the material (Figure S12), the reactivity toward isobutene cracking, propene coking,^15^ and the requirement of higher temperatures during the Cu ion exchange highlight the possibility of Cu gradients in the K-MOR series. This underestimation of species, such as [CuOH]^+^,of differing reactivity from Cu-oxo dimers at higher Cu loadings can explain the lack of volcanic behavior in the simulation data. Indeed, the combination with EPR, XAS, and reaction testing clearly suggests the presence of reactive and active monomeric Cu­[II] species participating in the partial oxidation of methane, likely in conjunction with dimeric Cu-oxo species.

## Conclusion

Density Functional Theory calculations imply that the specific siting of Al, induced by the presence of a mixture of Na and K cations, results in geometric distortions of the pore aperture, namely in the side pocket believed to be the influential site for active Cu-oxo species in the methane-to-methanol reaction. The more constrained environment in the case of Cu-MOR­(K) leads to the formation of more reducible and thereby reactive [CuOH]^+^, as seen through operando XAS and EPR studies, while also being predicted to form Cu-oxo dimers with more acute angles of higher reactivity based on Monte Carlo simulations. Varying the methane exposure time then confirmed the high productivity of the Cu-MOR­(K) system at low Cu loadings, achieving a productivity approaching the 0.5 MeOH/Cu limit at atmospheric pressure.

Thus, we can conclude that in the case of MOR the geometric distortions and placement of Al can result in the favorable formation of reactive Cu-oxo species, and this can be tuned by modifying the zeolite synthesis. This underscores the importance of synthetic strategies in designing Cu zeolites for alkane transformations and paving the way for the development of more efficient catalysts with improved performance.

## Supplementary Material



## Notes

Ion exchange isotherms along with Cu loadings of all investigated samples, framework models derived from DFT calculations, DRIFT spectra, MCR analysis of operando XAS data, additional EPR experiments, Monte Carlo simulations and details thereof, along with CH4 activation energy calculations, and EDX maps of Cu-exchanged samples (PDF)

## References

[1] Wang V. C. C., Maji S., Chen P. P. Y., Lee H. K., Yu S. S. F., Chan S. I. (2017). Alkane Oxidation: Methane Monooxygenases, Related Enzymes,
and Their Biomimetics. Chem. Rev..

[2] Solomon E. I., Heppner D. E., Johnston E. M., Ginsbach J. W., Cirera J., Qayyum M., Kieber-Emmons M. T., Kjaergaard C. H., Hadt R. G., Tian L. (2014). Copper Active Sites in Biology. Chem. Rev..

[3] Ross M. O., Rosenzweig A. C. (2017). A tale
of two methane monooxygenases. JBIC, J. Biol.
Inorg. Chem..

[4] Zhao T., Huang X., Cui R., Han W., Zhang G., Tang Z. (2023). Design of confined catalysts and
applications in environmental catalysis:
Original perspectives and further prospects. J. Cleaner Prod..

[5] Grommet A. B., Feller M., Klajn R. (2020). Chemical reactivity under nanoconfinement. Nat. Nanotechnol..

[6] Yang X., Xu Q. (2020). Encapsulating Metal
Nanocatalysts within Porous Organic Hosts. TRECHEM.

[7] Groothaert M. H., Smeets P. J., Sels B. F., Jacobs P. A., Schoonheydt R. A. (2005). Selective
Oxidation of Methane by the Bis­(*μ*-oxo)­dicopper
Core Stabilized on ZSM-5 and Mordenite Zeolites. J. Am. Chem. Soc..

[8] Kvande, K. A Study of Cu-Loaded SAPO-34 for the Direct Conversion of Methane to Methanol; University of Oslo, 2019.

[9] Newton M. A., Knorpp A. J., Pinar A. B., Sushkevich V. L., Palagin D., van Bokhoven J. A. (2018). On the Mechanism Underlying the Direct
Conversion of Methane to Methanol by Copper Hosted in Zeolites; Braiding
Cu K-Edge XANES and Reactivity Studies. J. Am.
Chem. Soc..

[10] Alayon E. M., Nachtegaal M., Ranocchiari M., van Bokhoven J. A. (2012). Catalytic
conversion of methane to methanol over Cu–mordenite. Chem. Commun..

[11] Pappas D. K., Martini A., Dyballa M., Kvande K., Teketel S., Lomachenko K. A., Baran R., Glatzel P., Arstad B., Berlier G. (2018). The Nuclearity of the Active Site for Methane to Methanol
Conversion in Cu-Mordenite: A Quantitative Assessment. J. Am. Chem. Soc..

[12] Newton M. A., Knorpp A. J., Sushkevich V. L., Palagin D., van Bokhoven J. A. (2020). Active
sites and mechanisms in the direct conversion of methane to methanol
using Cu in zeolitic hosts: a critical examination. Chem. Soc. Rev..

[13] Knorpp A. J., Pinar A. B., Newton M. A., Li T., Calbry-Muzyka A., van Bokhoven J. A. (2021). Copper-exchanged
large-port and small-port mordenite
(MOR) for methane-to-methanol conversion. RSC
Adv..

[14] Meier W. M. (1968). The crystal
structure of mordenite (ptilolite). Z. Kristallogr..

[15] Prodinger S., Capel Berdiell I., Cordero-Lanzac T., Bygdnes O. R., Solemsli B. G., Kvande K., Arstad B., Beato P., Olsbye U., Svelle S. (2023). Cation-induced
Speciation of Port-Size during Mordenite
Zeolite Synthesis. J. Mater. Chem. A.

[16] Sand L. B. (1968). Synthesis
of large-port and small-port mordenites. Mol.
Sieves.

[17] Fischer J. W. A., Brenig A., Klose D., van Bokhoven J. A., Sushkevich V. L., Jeschke G. (2023). Methane Oxidation over
Cu2+/[CuOH]+
Pairs and Site-Specific Kinetics in Copper Mordenite Revealed by Operando
Electron Paramagnetic Resonance and UV/Visible Spectroscopy. Angew. Chem., Int. Ed..

[18] Plessers D., Heyer A. J., Rhoda H. M., Bols M. L., Solomon E. I., Schoonheydt R. A., Sels B. F. (2023). Tuning Copper Active Site Composition
in Cu-MOR through Co-Cation Modification for Methane Activation. ACS Catal..

[19] Smeets P. J., Groothaert M. H., Schoonheydt R. A. (2005). Cu based zeolites: A UV–vis
study of the active site in the selective methane oxidation at low
temperatures. Catal. Today.

[20] Palagin D., Knorpp A. J., Pinar A. B., Ranocchiari M., van Bokhoven J. A. (2017). Assessing the relative stability
of copper oxide clusters
as active sites of a CuMOR zeolite for methane to methanol conversion:
size matters?. Nanoscale.

[21] Brezicki G., Zheng J., Paolucci C., Schlögl R., Davis R. J. (2021). Effect of the Co-cation on Cu Speciation
in Cu-Exchanged
Mordenite and ZSM-5 Catalysts for the Oxidation of Methane to Methanol. ACS Catal..

[22] Snyder B. E. R., Vanelderen P., Schoonheydt R. A., Sels B. F., Solomon E. I. (2018). Second-Sphere
Effects on Methane Hydroxylation in Cu-Zeolites. J. Am. Chem. Soc..

[23] Vanelderen P., Snyder B. E. R., Tsai M. L., Hadt R. G., Vancauwenbergh J., Coussens O., Schoonheydt R. A., Sels B. F., Solomon E. I. (2015). Spectroscopic
Definition of the Copper Active Sites in Mordenite: Selective Methane
Oxidation. J. Am. Chem. Soc..

[24] Wichterlová B., Sobalík Z., Dědeček J. (1997). Cu ion siting in high
silica zeolites. Spectroscopy and redox properties. Catal. Today.

[25] Dedecek J., Wichterlova B. (1994). Siting and Redox Behavior of Cu Ions
in CuH-ZSM-5 Zeolites.
Cu+ Photoluminescence Study. J. Phys. Chem..

[26] Dědeček J., Wichterlová B. (1999). Geometry of the Cu+ 540 nm luminescence centres in
zeolites. Phys. Chem. Chem. Phys..

[27] Smeets P. J., Woertink J. S., Sels B. F., Solomon E. I., Schoonheydt R. A. (2010). Transition-Metal
Ions in Zeolites: Coordination and Activation of Oxygen. Inorg. Chem..

[28] Vanelderen P., Vancauwenbergh J., Sels B. F., Schoonheydt R. A. (2013). Coordination
chemistry and reactivity of copper in zeolites. Coord. Chem. Rev..

[29] Delabie A., Pierloot K., Groothaert M. H., Schoonheydt R. A., Vanquickenborne L. G. (2002). The Coordination of CuII in Zeolites
– Structure
and Spectroscopic Properties. Eur. J. Inorg.
Chem..

[30] Fischer, J. W. A. ; Brenig, A. ; Klose, D. ; van Bokhoven, J. A. ; Sushkevich, V. L. ; Jeschke, G. Methane Oxidation over Cu2+/[CuOH]+ Pairs and Site-Specific Kinetics in Copper Mordenite Revealed by Operando Electron Paramagnetic Resonance and UV-Visible Spectroscopy. Angew. Chem., Int. Ed. 2023, 62, e202303574 10.1002/anie.202303574 37292054

[31] Wijerathne A., Sawyer A., Daya R., Paolucci C. (2024). Competition between
Mononuclear and Binuclear Copper Sites across Different Zeolite Topologies. JACS Au.

[32] Abdala P. M., Safonova O. V., Wiker G., van Beek W., Emerich H., Ja V. B., Sá J., Szlachetko J., Nachtegaal M., Van Bokhoven J. A. (2012). Scientific Opportunities for Heterogeneous
Catalysis Research at the SuperXAS and SNBL Beam Lines. Chimia.

[33] van
Beek W., Safonova O. V., Wiker G., Emerich H. (2011). SNBL, a dedicated beamline
for combined in situ X-ray diffraction, X-ray absorption and Raman
scattering experiments. Phase Transitions.

[34] Newville M. L. (2013). An Analysis
Package for XAFS and Related Spectroscopies. J. Phys. Conf. Ser..

[35] Höfer P., Grupp A., Nebenführ H., Mehring M. (1986). Hyperfine sublevel
correlation (hyscore) spectroscopy: a 2D ESR investigation of the
squaric acid radical. Chem. Phys. Lett..

[36] Stoll S., Schweiger A. (2006). EasySpin, a comprehensive software package for spectral
simulation and analysis in EPR. J. Magn. Reson..

[37] Kühne T. D., Iannuzzi M., Del Ben M., Rybkin V. V., Seewald P., Stein F., Laino T., Khaliullin R. Z., Schütt O. (2020). CP2K: An electronic structure and molecular
dynamics software package -Quickstep: Efficient and accurate electronic
structure calculations. J. Chem. Phys..

[38] Goedecker S., Teter M., Hutter J. (1996). Separable dual-space Gaussian pseudopotentials. Phys. Rev. B.

[39] Perdew J. P., Burke K., Ernzerhof M. (1996). Generalized
Gradient Approximation
Made Simple. Phys. Rev. Lett..

[40] Zhang Y., Wang H., Chen W., Zeng J., Zhang L., Wang H., E W. (2020). DP-GEN: A concurrent learning platform
for the generation of reliable deep learning based potential energy
models. Comput. Phys. Commun..

[41] Wang H., Zhang L., Han J., E W. (2018). DeePMD-kit:
A deep learning package
for many-body potential energy representation and molecular dynamics. Comput. Phys. Commun..

[42] Guo J., Sours T., Holton S., Sun C., Kulkarni A. R. (2024). Screening
Cu-Zeolites for Methane Activation Using Curriculum-Based Training. ACS Catal..

[43] Olson B., Hashmi I., Molloy K., Shehu A. (2012). Basin Hopping as a
General and Versatile Optimization Framework for the Characterization
of Biological Macromolecules. Adv. Artif. Intell..

[44] Wales D. J., Doye J. P. K. (1997). Global Optimization
by Basin-Hopping and the Lowest
Energy Structures of Lennard-Jones Clusters Containing up to 110 Atoms. J. Phys. Chem. A.

[45] Hjorth
Larsen A., Jørgen Mortensen J., Blomqvist J., Castelli I. E., Christensen R., Dułak M., Friis J., Groves M. N., Hammer B., Hargus C. (2017). The atomic simulation environment-a Python library for working with
atoms. J. Phys. Condens. Matter.

[46] Ch, B. ; Lb, M. ; Gies, H. ; Marler, B. Database of Disordered Zeolite Structures. http://www.iza-structure.org/databases/http://www.iza-structure.org/databases/. accessed September 2024.

[47] Prodinger S., Kvande K., Arstad B., Borfecchia E., Beato P., Svelle S. (2022). Synthesis–Structure–Activity
Relationship in Cu-MOR for Partial Methane Oxidation: Al Siting via
Inorganic Structure-Directing Agents. ACS Catal..

[48] Jangjou Y., Do Q., Gu Y., Lim L. G., Sun H., Wang D., Kumar A., Li J., Grabow L. C., Epling W. S. (2018). Nature
of Cu Active Centers in Cu-SSZ-13 and Their Responses to SO2 Exposure. ACS Catal..

[49] Luo J., Wang D., Kumar A., Li J., Kamasamudram K., Currier N., Yezerets A. (2016). Identification of two
types of Cu
sites in Cu/SSZ-13 and their unique responses to hydrothermal aging
and sulfur poisoning. Catal. Today.

[50] Kwak J. H., Zhu H., Lee J. H., Peden C. H. F., Szanyi J. (2012). Two different cationic
positions in Cu-SSZ-13?. Chem. Commun..

[51] Kwak J. H., Varga T., Peden C. H. F., Gao F., Hanson J. C., Szanyi J. (2014). Following the movement
of Cu ions in a SSZ-13 zeolite
during dehydration, reduction and adsorption: A combined *in
situ* TP-XRD, XANES/DRIFTS study. J.
Catal..

[52] Solemsli B. G., Berdiell I. C., Prodinger S., Kvande K., Deplano G., Olsbye U., Beato P., Bordiga S., Svelle S. (2024). Reactivity
of methoxy species towards methylation and oligomerization in Cu-zeolite
systems. Catal. Today.

[53] Pappas D. K., Borfecchia E., Dyballa M., Pankin I. A., Lomachenko K. A., Martini A., Signorile M., Teketel S., Arstad B., Berlier G. (2017). Methane
to Methanol: Structure–Activity Relationships
for Cu-CHA. J. Am. Chem. Soc..

[54] Borfecchia E., Lomachenko K. A., Giordanino F., Falsig H., Beato P., Soldatov A. V., Bordiga S., Lamberti C. (2015). Revisiting the nature
of Cu sites in the activated Cu-SSZ-13 catalyst for SCR reaction. Chem. Sci..

[55] Alayon E. M. C., Nachtegaal M., Bodi A., van Bokhoven J. A. (2014). Reaction
Conditions of Methane-to-Methanol Conversion Affect the Structure
of Active Copper Sites. ACS Catal..

[56] Martini A., Borfecchia E. (2020). Spectral Decomposition
of X-ray Absorption Spectroscopy
Datasets: Methods and Applications. Crystals.

[57] Jaumot J., de Juan A., Tauler R. (2015). MCR-ALS GUI
2.0: New features and
applications. Chemom. Intell. Lab. Syst..

[58] Kvande K., Garetto B., Deplano G., Signorile M., Solemsli B. G., Prodinger S., Olsbye U., Beato P., Bordiga S., Svelle S. (2023). Understanding C–H
activation in light alkanes over Cu-MOR zeolites by coupling advanced
spectroscopy and temperature-programmed reduction experiments. Chem. Sci..

[59] Bregante D. T., Wilcox L. N., Liu C., Paolucci C., Gounder R., Flaherty D. W. (2021). Dioxygen Activation
Kinetics over Distinct Cu Site
Types in Cu-Chabazite Zeolites. ACS Catal..

[60] Lomachenko K. A., Martini A., Pappas D. K., Negri C., Dyballa M., Berlier G., Bordiga S., Lamberti C., Olsbye U., Svelle S. (2019). The impact of reaction conditions and material composition
on the stepwise methane to methanol conversion over Cu-MOR: An *operando* XAS study. Catal. Today.

[61] Sushkevich V. L., Palagin D., Ranocchiari M., van Bokhoven J. A. (2017). Selective
anaerobic oxidation of methane enables direct synthesis of methanol. Science.

[62] Moreno-González M., Blasco T., Góra-Marek K., Palomares A. E., Corma A. (2014). Study of propane oxidation
on Cu-zeolite catalysts by in-situ EPR
and IR spectroscopies. Catal. Today.

[63] Chao C., Lunsford J. H. (1972). EPR Study of Copper
(II) Ion Pairs in Y-Type Zeolites. J. Chem.
Phys..

[64] Bruzzese P. C., Salvadori E., Jäger S., Hartmann M., Civalleri B., Pöppl A., Chiesa M. (2021). 17O-EPR determination of the structure
and dynamics of copper single-metal sites in zeolites. Nat. Commun..

[65] Bruzzese P. C., Salvadori E., Civalleri B., Jäger S., Hartmann M., Pöppl A., Chiesa M. (2022). The Structure of Monomeric
Hydroxo-CuII Species in Cu-CHA. A Quantitative Assessment. J. Am. Chem. Soc..

[66] Larsen S. C., Aylor A., Bell A. T., Reimer J. A. (1994). Electron Paramagnetic
Resonance Studies of Copper Ion-Exchanged ZSM-5. J. Phys. Chem..

[67] Sushkevich V. L., Smirnov A. V., van Bokhoven J. A. (2019). Autoreduction
of Copper in Zeolites:
Role of Topology, Si/Al Ratio, and Copper Loading. J. Phys. Chem. C.

[68] Wulfers M. J., Teketel S., Ipek B., Lobo R. F. (2015). Conversion of methane
to methanol on copper-containing small-pore zeolites and zeotypes. Chem. Commun..

[69] Alayon E. M. C., Nachtegaal M., Bodi A., Ranocchiari M., van Bokhoven J. A. (2015). Bis­(μ-oxo)
versus mono­(μ-oxo)­dicopper cores
in a zeolite for converting methane to methanol: an in situ XAS and
DFT investigation. Phys. Chem. Chem. Phys..

[70] Vanelderen P., Vancauwenbergh J., Tsai M. L., Hadt R. G., Solomon E. I., Schoonheydt R. A., Sels B. F. (2014). Spectroscopy and Redox Chemistry
of Copper in Mordenite. ChemPhyschem.

[71] Rhoda H. M., Plessers D., Heyer A. J., Bols M. L., Schoonheydt R. A., Sels B. F., Solomon E. I. (2021). Spectroscopic Definition of a Highly
Reactive Site in Cu-CHA for Selective Methane Oxidation: Tuning a
Mono-μ-Oxo Dicopper­(II) Active Site for Reactivity. J. Am. Chem. Soc..

[72] Mahyuddin M. H., Staykov A., Shiota Y., Miyanishi M., Yoshizawa K. (2017). Roles of Zeolite Confinement and
Cu–O–Cu
Angle on the Direct Conversion of Methane to Methanol by [Cu2­(μ-O)]­2
± Exchanged AEI, CHA, AFX, and MFI Zeolites. ACS Catal..

[73] Wilcox L. N., Rebolledo-Oyarce J., Mikes A. D., Wang Y., Schneider W. F., Gounder R. (2024). Structure and Reactivity of Binuclear
Cu Active Sites
in Cu-CHA Zeolites for Stoichiometric Partial Methane Oxidation to
Methanol. ACS Catal..

[74] Narsimhan K., Iyoki K., Dinh K., Román-Leshkov Y. (2016). Catalytic
Oxidation of Methane into Methanol over Copper-Exchanged Zeolites
with Oxygen at Low Temperature. ACS Cent. Sci..

[75] Chen H., Matsuoka M., Zhang J., Anpo M. (2004). The reduction behavior
of the Cu ion species exchanged into Y zeolite during the thermovacuum
treatment. J. Catal..

[76] Sushkevich V. L., Artsiusheuski M., Klose D., Jeschke G., van Bokhoven J. A. (2021). Identification
of Kinetic and Spectroscopic Signatures of Copper Sites for Direct
Oxidation of Methane to Methanol. Angew. Chem.,
Int. Ed..

[77] Han P., Zhang Z., Chen Z., Lin J., Wan S., Wang Y., Wang S. (2021). Critical Role of Al Pair Sites in
Methane Oxidation to Methanol on Cu-Exchanged Mordenite Zeolites. Catalysts.

[78] Zhang, H. ; Han, P. ; Wu, D. ; Du, C. ; Zhao, J. ; Zhang, K. H. ; Lin, J. ; Wan, S. ; Huang, J. ; Wang, S. Confined Cu-OH single sites in SSZ-13 zeolite for the direct oxidation of methane to methanol Nat. Commun. 14 7705 10.1038/s41467-023-43508-4. PMC10673993 38001068

[79] Becher J., Sanchez D. F., Doronkin D. E., Zengel D., Meira D. M., Pascarelli S., Grunwaldt J. D., Sheppard T. L. (2021). Chemical gradients
in automotive Cu-SSZ-13 catalysts for NOx removal revealed by operando
X-ray spectrotomography. Nat. Catal..

