# Supplementary material for: Influence of Aluminum Distribution in Cu-MOR Systems on Methane-to-Methanol Conversion: A Combined Experimental and Theoretical Study
Source: J Phys Chem C Nanomater Interfaces. 2025 Oct 18;129(44):20044–56. doi: 10.1021/acs.jpcc.5c06045 (PMC12598874; doi:10.1021/acs.jpcc.5c06045)
Supplement: Supplementary file 1 [file jp5c06045_si_001.pdf]

Supporting Information

Influence of Aluminum Distribution in Cu-MOR Systems on Methane-to-Methanol Conversion: A Combined Experimental and Theoretical Study

Authors: *Peter N. Njoroge*[†#], *Bjørn Gading Solemsli*[†#], *Asanka Wijerathne*[±], *Izar Capel Berdiell*[†], *Agnieszka Seremak*[†], *Mario Chiesa*[‡], *Yu-Kai Liao*[‡], *Beatrice Garetto*[‡], *Nishant Patel*[‡], *Karoline Kvande*[⁎], *Elisa Borfecchia*[‡], *Christopher Paolucci*[±], *Unni Olsbye*[†], *Pablo Beato*[§], *Stian Svelle*[†], *Sebastian Prodinger*[§†*]

†Centre for Materials Science and Nanotechnology (SMN), Department of Chemistry, University of Oslo, 1033 Blindern, 0315 Oslo, Norway

[±]Department of Chemical Engineering, University of Virginia, Charlottesville, VA 22903, United States of America

[⁎]Department of Chemical and Biomolecular Engineering, University of California, Berkeley, CA 94720, United States of America

‡Department of Chemistry, NIS Centre and INSTM Reference Centre, University of Turin, via P. Giuria 7, 10125 Turin, Italy

[§]Topsoe A/S, Haldor Topsøes Allé 1, 2800 Kongens Lyngby, Denmark

e-mail: sepr@topsoe.com

Table of Contents

1.Authors contribution 3

2.Reaction protocol 4

Scheme S1 4

3.Density functional theory calculations 4

Figure S1: 4

Table S1 5

Table S2: 5

Figure S2 6

4.Copper incorporation and MTM testing 7

Table S3 7

Figure S3 7

5.Drifts 8

Figure S4 8

6.X-ray absorption spectroscopy 9

Figure S5: 9

7. X-ray Diffraction 10
Figure S6: 10
8.Electron paramagnetic resonance 10
Figure S7: 11
Figure S8: 11
Figure S9 12
9.Monte Carlo simulations 13
Table S4 13
Figure S10: 13
10.Optimize initial, final and transition states for CH_4 activation energy calculations 13
Figure S11: 14
11. Backscattering and EDX mapping of materials. 14
Figure S12: 15
12.References 15

1.Authors contribution

Peter N. Njoroge
Investigation – testing
Formal Analysis
Visualization - support
Writing – review & editing - main

Bjørn Gading Solemsli
Investigation – testing, XAS, DRIFTS
Formal Analysis
Visualization
Writing – original draft

Asanka Wiejeranthe
Investigation – MC simulations
Formal analysis
Visualization
Writing – review & editing

Izar Capel Berdiell
Investigation – XRD
Formal analysis
Writing – review & editing

Mario Chiesa
Investigation – EPR
Formal analysis
Writing – review & editing support

Yu-Kai Liao
Investigation - EPR

Agnieszka Seremak
Investigation – DFT geometry optimization
Formal analysis
Writing – review & editing support

Beatrice Garetto
Investigation – XAS
Formal analysis
Writing – review & editing

Nishant Patel
Investigation – XAS
Writing – review & editing support

Karoline Kvande
Writing – review & editing support

Elisa Borfecchia
Investigation - XAS
Writing – review & editing support
Supervision – BG, NP

Christopher Paolucci
Writing – review & editing support
Methodology – MC simulations
Supervision - AW

Unni Olsbye
Writing – review & editing support
Supervision – PNN
Funding acquisition
Resources

Pablo Beato
Writing – review & editing support

Stian Svelle
Writing – review & editing support
Supervision – BGS, KK
Funding acquisition
Resources

Sebastian Prodinger
Conceptualization
Methodology
Investigation – material preparation
Formal Analysis
Supervision – PNN, BGS
Writing – review & editing

2.Reaction protocol

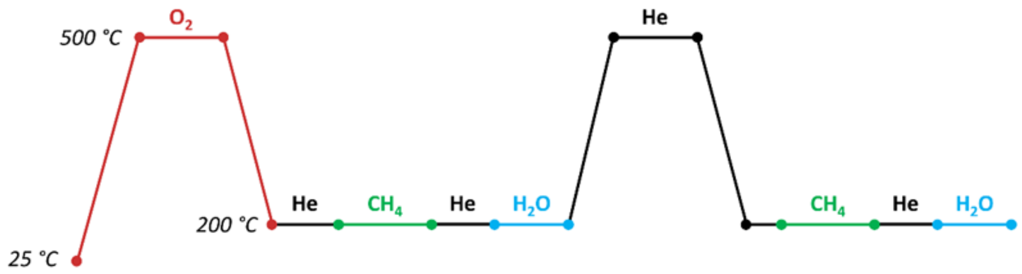

Scheme S1: Temperature profile depicting the reaction protocol. The temperature ramp rate was kept at 10 °C min^{-1}.

3.Density functional theory calculations

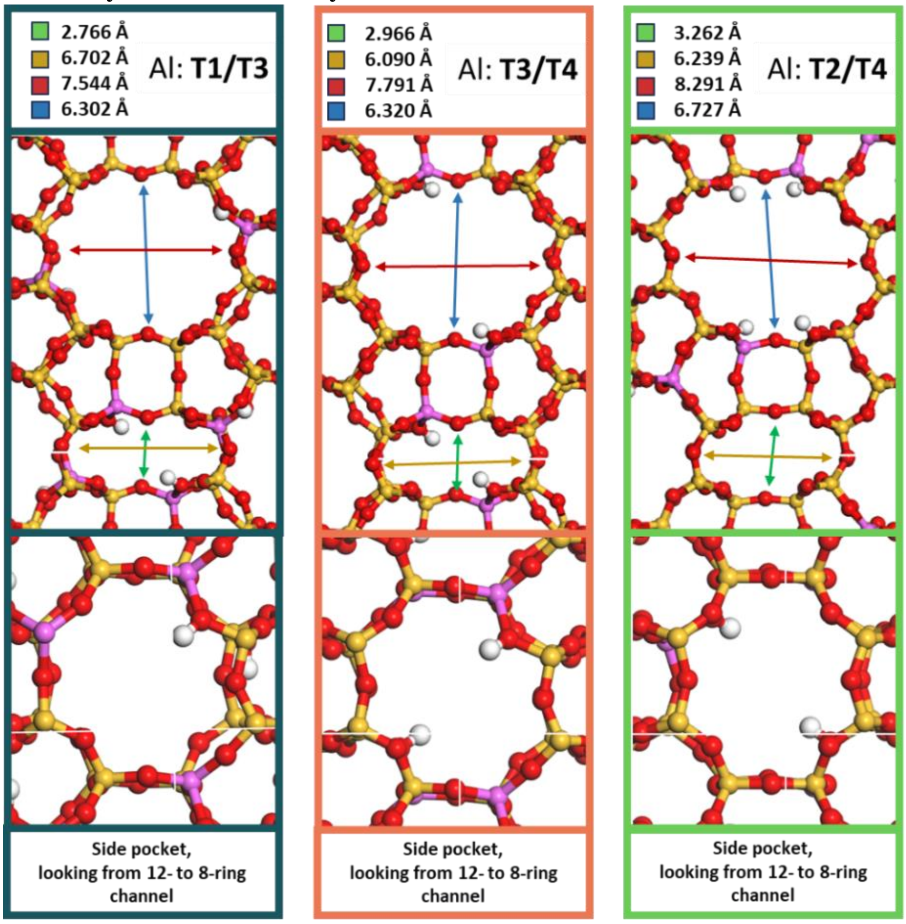

Figure S1: Models of three of the Al T-pairs for MOR (Si/Al=5). The distances used to determine the 12-ring and compressed 8-ring pore dimensions are shown in green, yellow, red, and blue.

		T1/T3	T1/T3*	T2/T3	T3/T4	T1/T2	T2/T4	T1/T4	T3/T4*
	Energy (kJ/mol)	0.00	34.96	83.13	58.96	82.30	107.07	144.70	185.79
Channels	12-ring (Å)	6.302	6.261	6.697	11841	6.371	6.727	6.218	6.760
		7.544	7.496	7.432	7.791	7.994	8.291	8.046	7.931
	8-ring (Å)	6.702	6.698	6.090	6.518	7.285	6.239	6.331	6.676
		2.766	2.558	2.851	2.699	3.176	3.262	3.041	3.033
Side pocket openings	12-ring (Å)	4.801	4.309	3.694	4.789	4.853	24167	35125	4.896
		3.644	4.821	3.569	3.376	3.569	4.915	4.866	4.094
		3.685	3.606	4.866	3.873	3.515	4.280	4.137	3.738
		4.215	3.525	4.494	4.689	4.373	3.941	3.901	3.970
	Area (Å²)	***13.144***	***12.980***	***13.564***	***13.734***	***13.058***	***13.848***	***13.960***	***13.687***
	8-ring (Å)	4.801	12145	4.866	4.789	4.853	4.915	4.866	4.896
		3.312	4.771	3.463	3.089	3.502	3.852	3.437	4.025
		45748	4.821	3.592	3.793	3.951	3.403	3.959	3.859
		4.807	3.391	4.408	4.879	4.361	3.916	4.284	3.948
	Area (Å²)	***14.471***	***14.713***	***13.088***	***13.445***	***13.636***	***12.702***	***13.439***	***13.736***
	12/8-ratio	0.91	0.88	0.96	1.02	0.96	1.09	1.04	1.00

Table S1: Distances derived for the pores in MOR with either Si/Al-ratio of 5. The color of the numbers indicates the arrow on the models in Figure S1. For the side pocket openings, the average of the O-O bond lengths was used to calculate the area of a circle (for simplicity).

		T3/T4	T1/T3	T1/T2	T2/T4
	Energy (kJ/mol)	0.00	-19.70	-26.10	-30.47
Channels	12-ring (Å)	6.22	6.10	6.35	6.49
		7.323	7.598	8.034	8.237
	8-ring (Å)	5.642	5.561	5.391	6.156
		2.55	2.803	3.262	2.984
Side pocket openings	12-ring (Å)	3.781	3.775	3.638	3.841
		4.837	3.527	3.467	4.845
		4.129	4.810	4.809	3.994
		4.066	4.325	4.344	4.304
	Area (Å²)	***13.876***	***13.262***	***12.975***	***14.160***
	8-ring (Å)	3.437	3.982	4.809	3.392
		3.835	4.630	3.270	3.340
		4.837	4.810	3.503	4.845
		3.987	3.371	4.548	4.575
	Area (Å²)	***12.718***	***13.843***	***12.771***	***12.806***
	12/8-ratio	1.09	0.96	1.02	1.11

Table S2: Distances derived for the pores in MOR with either Si/Al-ratio of 11. The color of the numbers indicates the arrow on the models in Figure S1. For the side pocket openings, the average of the O-O bond lengths was used to calculate the area of a circle (for simplicity).

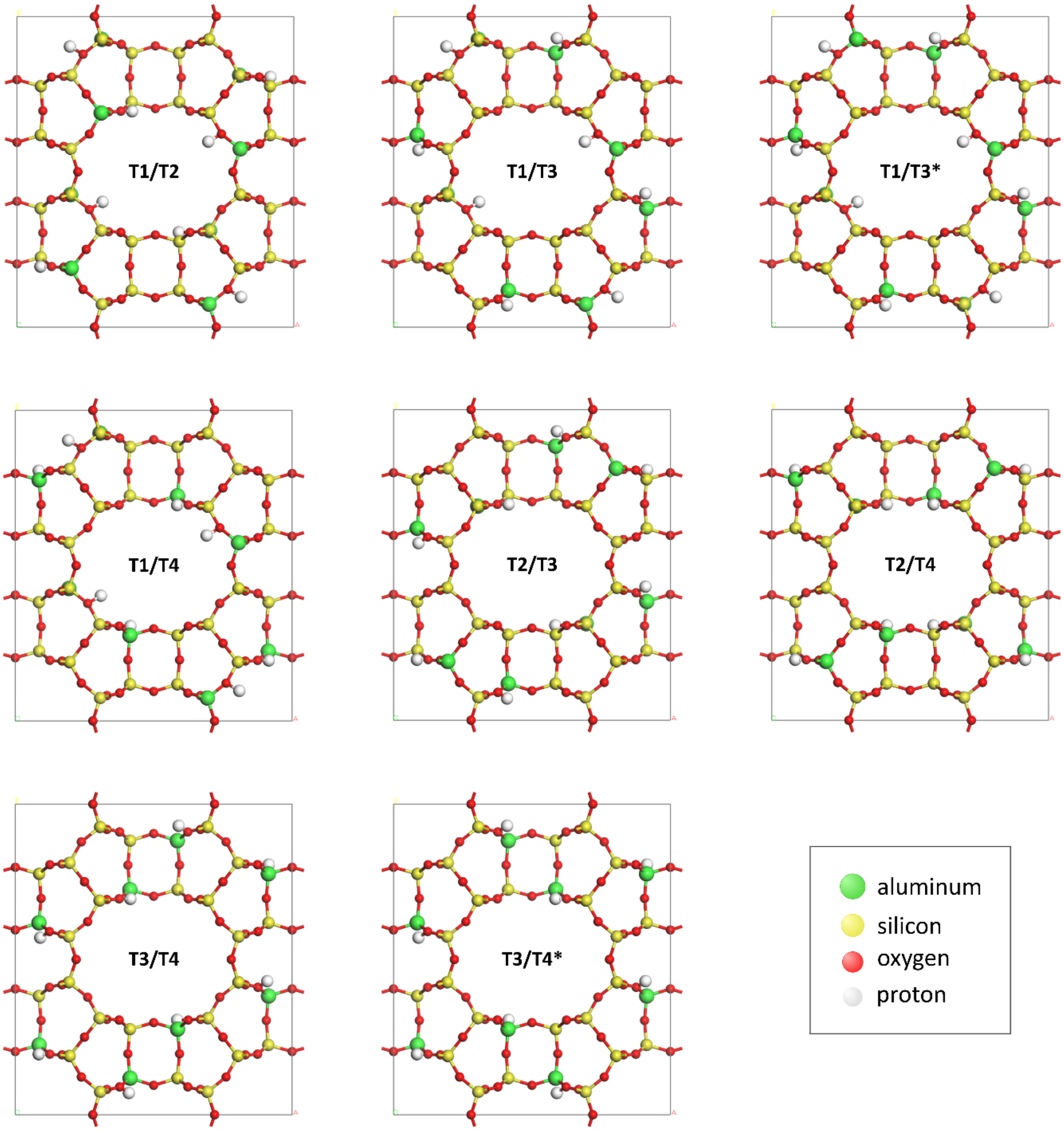

Figure S2: Various T site combinations considered for this study.

4.Copper incorporation and MTM testing

Sample	Molarity of copper acetate	Si/Al ratio	Cu/Al ratio	Copper loading (µmol $g_{zeolite}^{-1}$)	Methanol Yield (µmol $g_{zeolite}^{-1}$)
**0.02Cu-MOR(K)**	0.0006$^{\Delta}$	7.3	0.02	31 (±2.2)	3.8
**0.04Cu-MOR(K)**	0.02^{RT}	7.3	0.04	92(±6.5)	24.2
**0.07Cu-MOR(K)a**	0.01$^{\Delta}$	7.3	0.07	146.5 (±10.4)	68.4
0.07Cu-MOR(K)b	0.01$^{\Delta}$	7.3	0.07	147 (±10.4)	67.7
**0.07Cu-MOR(K)c**	0.02$^{\Delta}$	7.3	0.07	158 (±11.2)	68.1
**0.09Cu-MOR(K)**	0.02$^{\Delta+RT}$	7.3	0.09	223 (±15.9)	92.7
**0.17Cu-MOR(K)**	0.01$^{\Delta+\Delta}$	7.3	0.17	397 (±28.2)	108.1
**0.04Cu-MOR(Na)**	0.0025^{RT}	7.5	0.04	80 (±5.7)	11.5
**0.07Cu-MOR(Na)a**	0.005^{RT}	7.5	0.07	145 (±10.3)	33.5
0.07Cu-MOR(Na)b	0.005^{RT}	7.5	0.07	148 (±10.5)	37.3
**0.07Cu-MOR(Na)c**	0.005^{RT}	7.5	0.07	157 (±11.2)	49.6
**0.12Cu-MOR(Na)**	0.01^{RT}	7.5	0.12	266 (±18.9)	92.8
**0.13Cu-MOR(Na)**	0.01^{RT}	7.5	0.13	306 (±21.8)	104.7
**0.19Cu-MOR(Na)**		7.5	0.19	398.5 (±28.3)	114.3

Table S3: Material properties of each of the samples. $^{\Delta}$Cu(acetate)$_2$ ion exchange performed at 60 °C. ^{RT} Cu(acetate)$_2$ ion exchange performed at ambient temperature. $^{+}$ ion exchange performed twice. Si/Al and Al content were determined from MP-AES and error margins are reported from performing multiple analyses across different batches. The average error margin for M+/Al content is ca. 5%. Samples discussed at length in this contribution are highlighted by italic font.

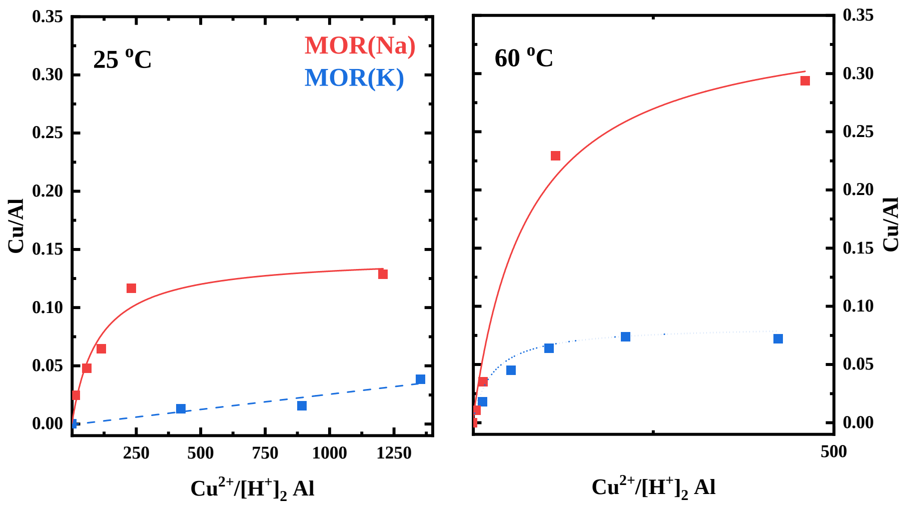

Figure S3: Exchange isotherms on the MOR(K) and the MOR(Na), from LIE performed at 25 °C and 60 °C, with fitted isotherm-curves. On the 60 °C isotherms, the last point of the MOR(K) series is not included in the isotherm, as it was exchanged twice.

5.Drifts

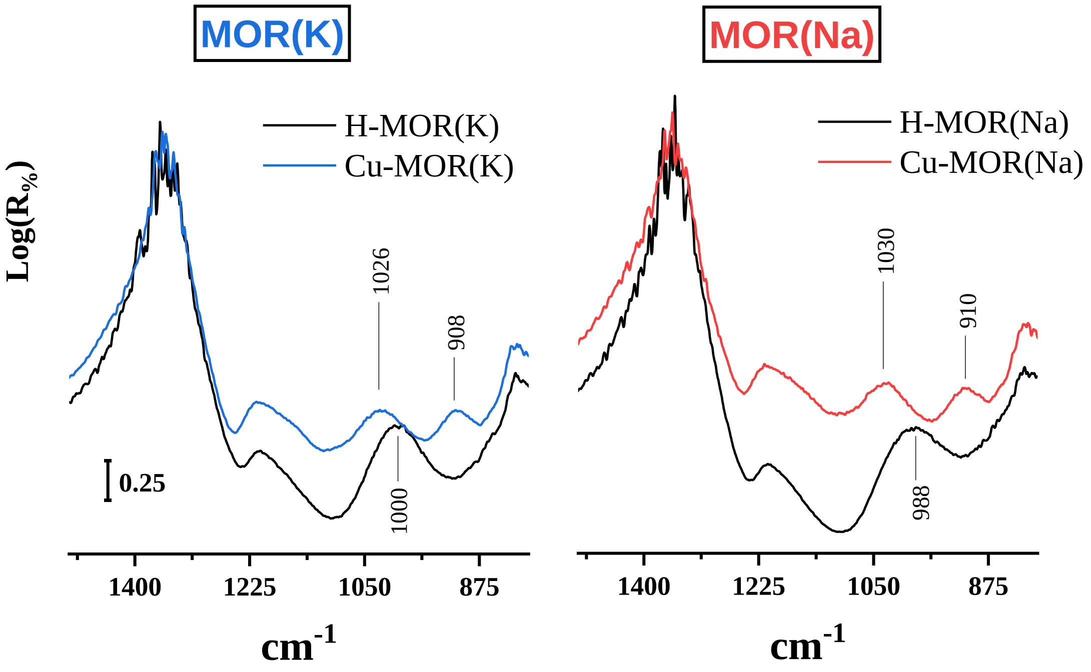

Figure S4: DRIFTS spectra of the MOR(K) and MOR(Na) materials for the proton-form (solid lines) and copper-form (dotted lines). For the copper-form the Cu-MOR(K) and Cu-MOR(Na) was used. All materials were calcined for 2 hours at 500 °C in pure O_2 before collecting at 300 °C. KBr was used as the background spectra.

6.X-ray absorption spectroscopy

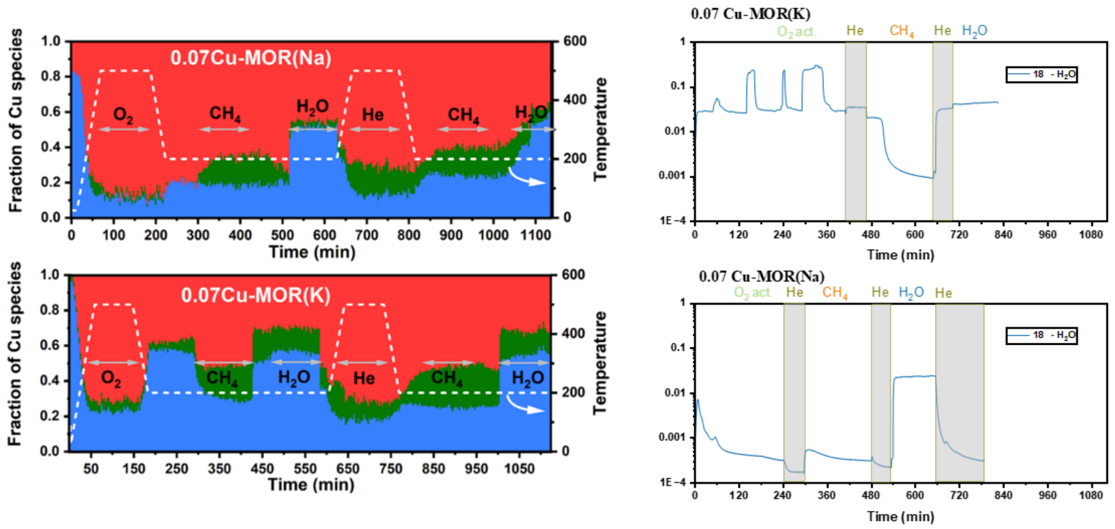

Figure S5: Temperature profile depicting the rection protocol with methane followed by XAS. The temperature ramp rate was kept at 10 °C min^{-1}. The high fraction of Cu-hyd in the Cu-MOR(K) samples is due to water contaminations, hydrating the sample. The pure spectra used for the MCR analysis is depicted on Figure 3E in the main text: red is Cu[II], green is Cu[I] and blue is Cu-hyd. The MS data collected during the in situ XAS measurements tracking the water signal.

7. X-ray Diffraction

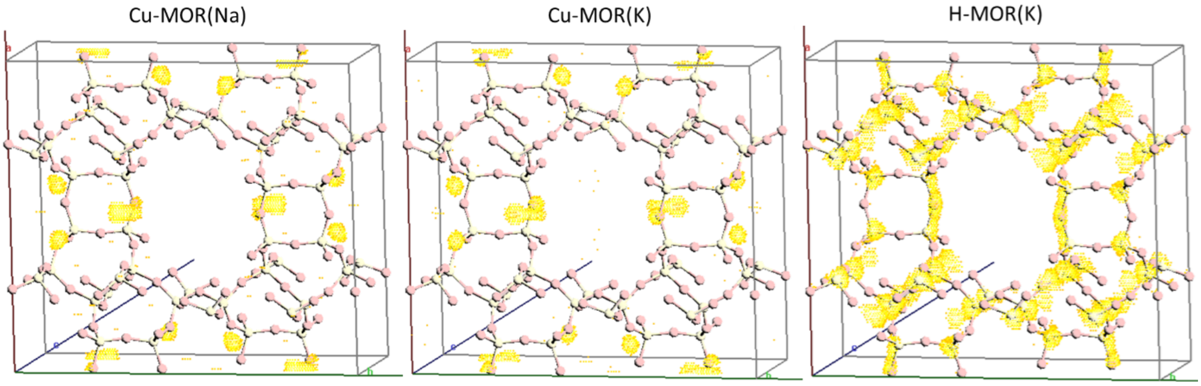

Figure S6: Difference Fourier map between identical structure factors calculated of a pure silica MOR empty framework and the dry Cu-MOR(Na)-left and the dry Cu-MOR(K)-center, as well as the H-MOR(K)-left.

8.Electron paramagnetic resonance

X-band (microwave frequency 9.45 GHz) CW-EPR spectra were acquired at 77 K on a Bruker EMX spectrometer equipped with a ER 4119 HS cylindrical cavity and at 10 K on a Bruker ELEXYS 580 spectrometer. In both cases, modulation frequency of 100 kHz, a modulation amplitude of 1 mT, and a microwave power of 2 mW were adopted. Pulse EPR measurements were performed at 10 K at X-band (microwave frequency 9.75 GHz) on a Bruker ELEXYS 580 spectrometer equipped with a cryogen-free cryostat from Cryogenics.

X-band electron-spin-echo (ESE) detected EPR spectra were acquired with the pulse sequence *π/2-τ-π-τ-echo*. The pulse lengths of $t_{\pi/2}$ = 16 ns, t_{π} = 32 ns and a τ value of 200 ns were used in conjunction of a shot repetition time of 3.55 KHz. For comparison with CW-EPR spectra (Figure S7) 2D experiments were performed by recording the ESE spectrum as a function of increasing τ and magnetic field. The τ value was incremented in steps of 16 ns starting from 100 ns, while the magnetic field was stepped in intervals of 0.5 mT. The projection sum of the ESE decay traces against the resonant magnetic field provides the EPR absorption spectrum free of distortions due to ESEEM modulation effects. The first derivative of the ESE spectrum was obtained using the pseudo modulation algorithm with a modulation amplitude of 1 mT.

Phase memory times (T_m) were measured using the Hahn Echo sequence upon increasing the interpulse delay τ starting from τ = 100 ns . The pulse lengths were $t_{\pi/2}$ = 40 ns and t_{π} = 80 ns. Spin−lattice relaxation times (T_1) were measured using the standard inversion recovery sequence (π−t_d−π/2−τ−π−τ−echo), with $t_{\pi/2}$ = 16 ns.

X-band Hyperfine Sublevel Correlation (HYSCORE)[1] spectroscopy measurements were carried out with the standard pulse sequence *π/2-τ-π/2-*t_1*-π-*t_2*- π/2- τ-echo*, employing a eight-step phase cycle for deleting unwanted echoes. Pulse lengths $t_{\pi/2}$ = 16 ns, t_{π} = 32 ns and a shot repetition time of 1.77 KHz were used. The increment of the time intervals t_1 and t_1 was 16 ns, starting from 80 to 2704 ns giving a data matrix of 170x170. Two τ values (104ns and 140 ns) were used. The corresponding spectra were added together after Fourier transform to eliminate blind spot features. The time traces of HYSCORE spectra were baseline corrected with a third-order polynomial, apodized with a Hamming window and zero-filled to 2048 points. After 2D Fourier transformation, the absolute-value spectra were calculated.

All the EPR spectra were simulated by using the Easyspin package[2] running in Matlab.

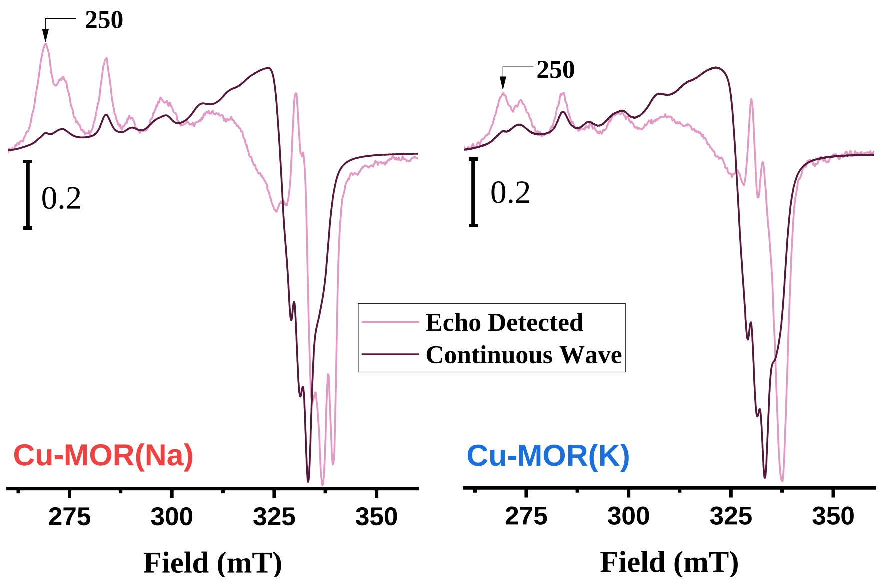

Figure S7: CW-EPR spectra and ESE spectra of Cu-MOR(Na) (left) and Cu-MOR(K) (right) after oxygen activation. CW EPR is depicted in marron and ESE EPR in pink. The ESE spectra were obtained as a 2D echo decay experiment as a function of the resonant magnetic field in order to avoid artifacts due to echo modulation effects. The resolution in the magnetic field was of 0.5 mT. All spectra were recorded at 10 K.

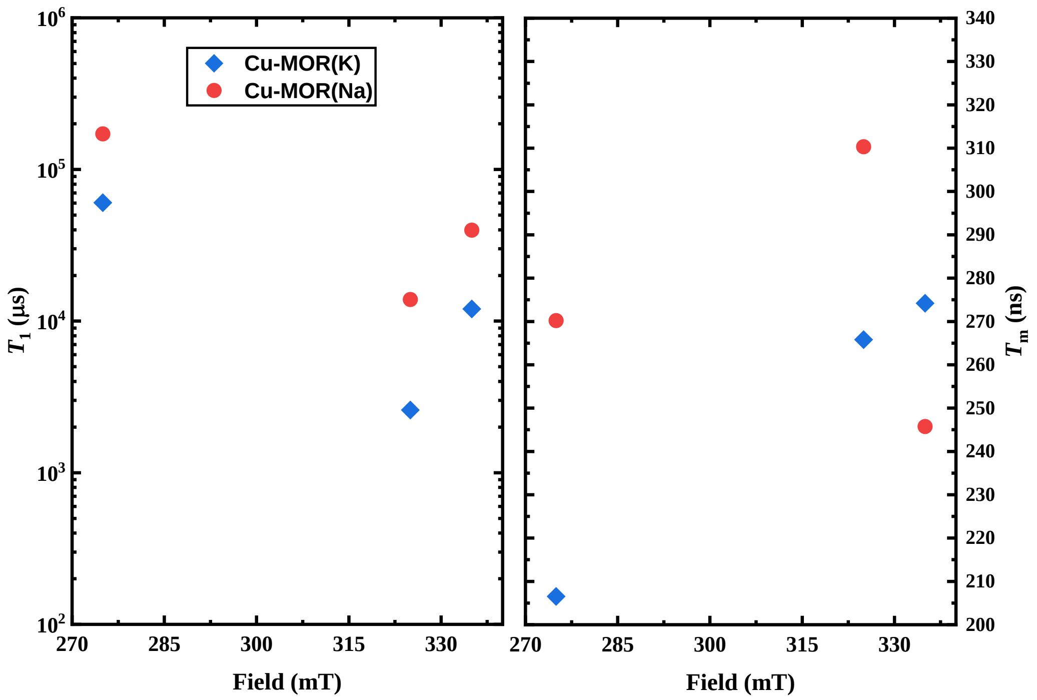

Figure S8: T_1 and T_m relaxation times measured at different magnetic field positions at 10 K.

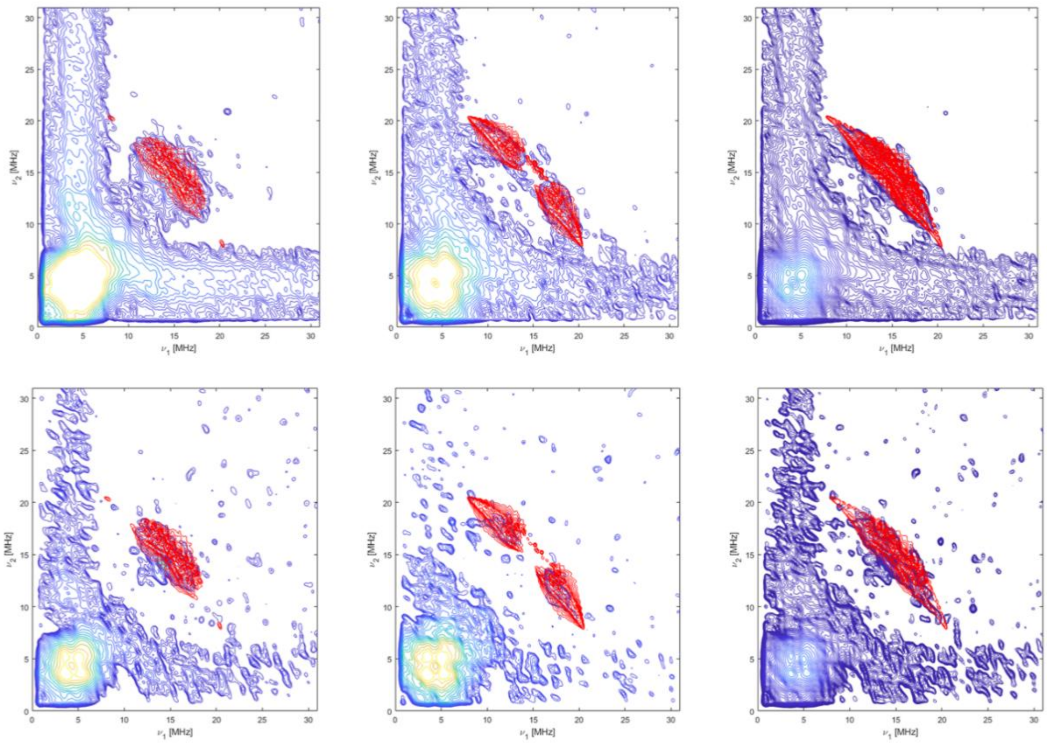

Figure S9: X-band ^{1}H HYSCORE spectra recorded at T=4 K and at a magnetic field corresponding to the maximum echo intensity and coinciding with $g_{\perp}$ (B_0=325 mT) MOR(Na) top and MOR(K) bottom. The simulation of the ^{1}H HYSCORE ridge is superimposed in red to the experimental spectrum. The spin-Hamiltonian parameters employed in the simulation are listed in Table x and coincide with those reported in ref. 42 of the main text for $[CuOH]^+$ species in Cu-CHA.

9.Monte Carlo simulations

	T1	T2	T3	T4
Cu-MOR(K)	0	41	0	59
Cu-MOR(Na)	55	0	45	0

Table S4: Percentage of Al in each T site used for simulations for Cu-MOR(K), and Cu-MOR(Na)

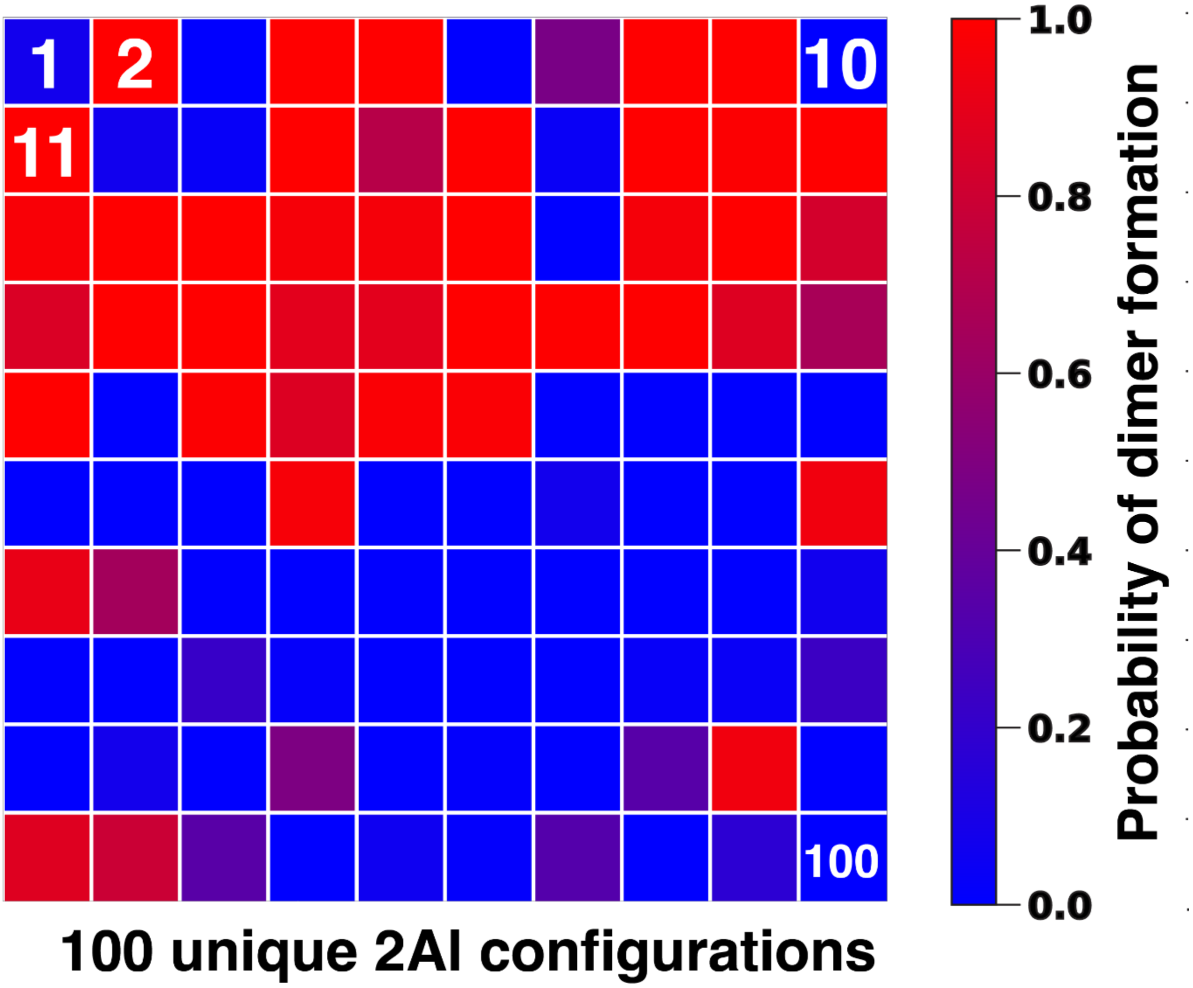

Figure S10: Dimer formation probabilities calculated at T=773 K, P_{H2O}=10^{-8} kPa, and P_{O2} = 20 kPa for 100 Al configurations in MOR. The numbers denoting each dimer type follow the same order convention of free energies (evaluated at T=773 K, P_{H2O}=10^{-8} kPa, and P_{O2} = 20 kPa) as our previous work[3].

10.Optimize initial, final and transition states for CH_4 activation energy calculations

To do NEB calculations for C-H activation energy estimations, first we need to create initial guesses for the structures of reactant, transition state, and product. The reactant is CH_4 adsorbed on Z_2Cu_2O, and the product has CH_4 with one C-H bond cleaved (CH_3 radical) and the cleaved H atom bonded to the O atom in the Cu dimer (Z_2Cu_2O-H). By mimicking the initial, transition, and product state reported for Cu dimers in MOR in a previous study[4], we created initial guesses by placing the required atoms. We created 100 initial guesses for CH_4 adsorbed on each Z_2Cu_2O dimer by randomly placing CH_4 near Z_2Cu_2O dimer while maintaining O-H distance of 1.5 Å and rejecting structures with overlapping atoms where distance between framework atoms and adsorbed CH_4 less than 1.0 Å. Then we minimized the energy of each initial structure using the DeePMD force field. Next, we selected the minimum energy structure with CH_4 adsorbed on each Z_2Cu_2O dimer and created the corresponding transition and product state for C-H activation reaction by changing C-H distance and O-H distance of the initial structure (Figure S11).

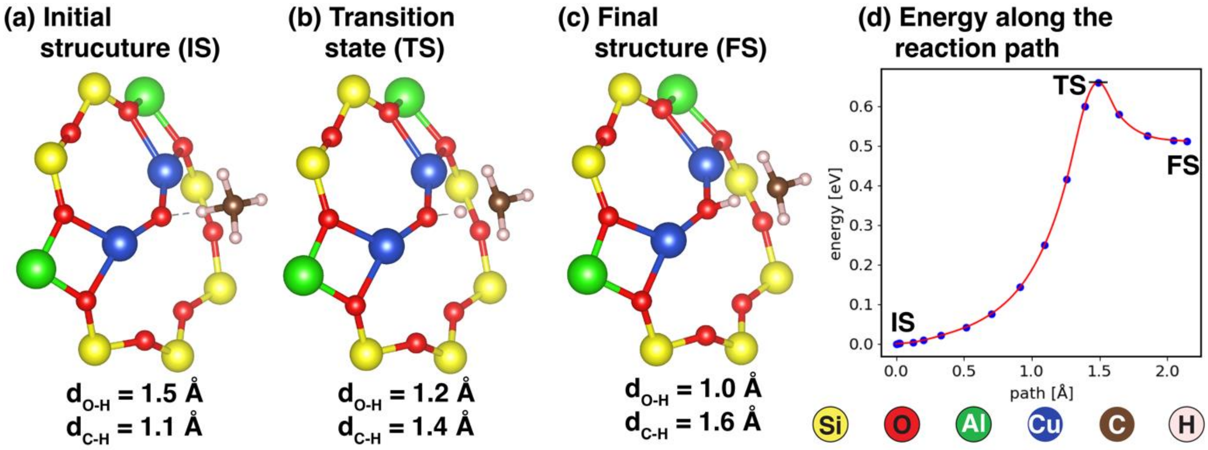

Figure S11: Structure sampling for dimer configuration 3 with corresponding O-H and C-H distances for, (a) initial structure with CH_4 adsorbed on the dimer, (b) transition state with C-H bond dissociation and O-H bond formation, and (c) final structure with fully dissociated C-H bond. The potential energy variation along the reaction path, calculated from NEB method using the pre-trained DeePMD force field is shown in (d)

11. Backscattering and EDX mapping of materials.

Backscattered electron imaging (BSE) and Energy Dispersive X-ray Mapping were performed on the samples to investigate the distribution of Cu within the samples. No evidence of nanoparticles formation is observed using the BSE technique across the different materials. While the EDX maps exhibited brighter spots in the higher loading samples compared to lower loading, there observations do not provide conclusive evidence for clear gradients in Cu distribution across the samples.

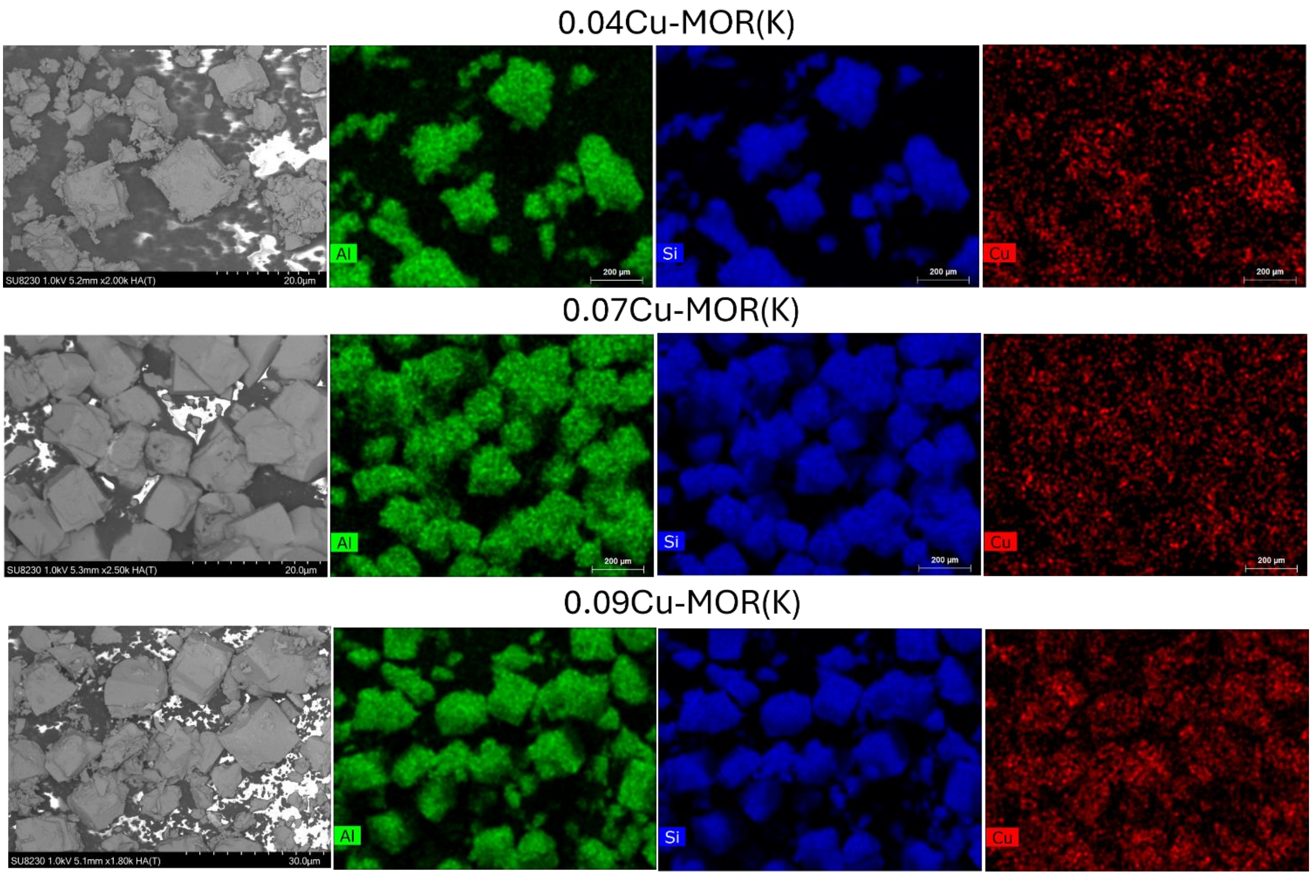

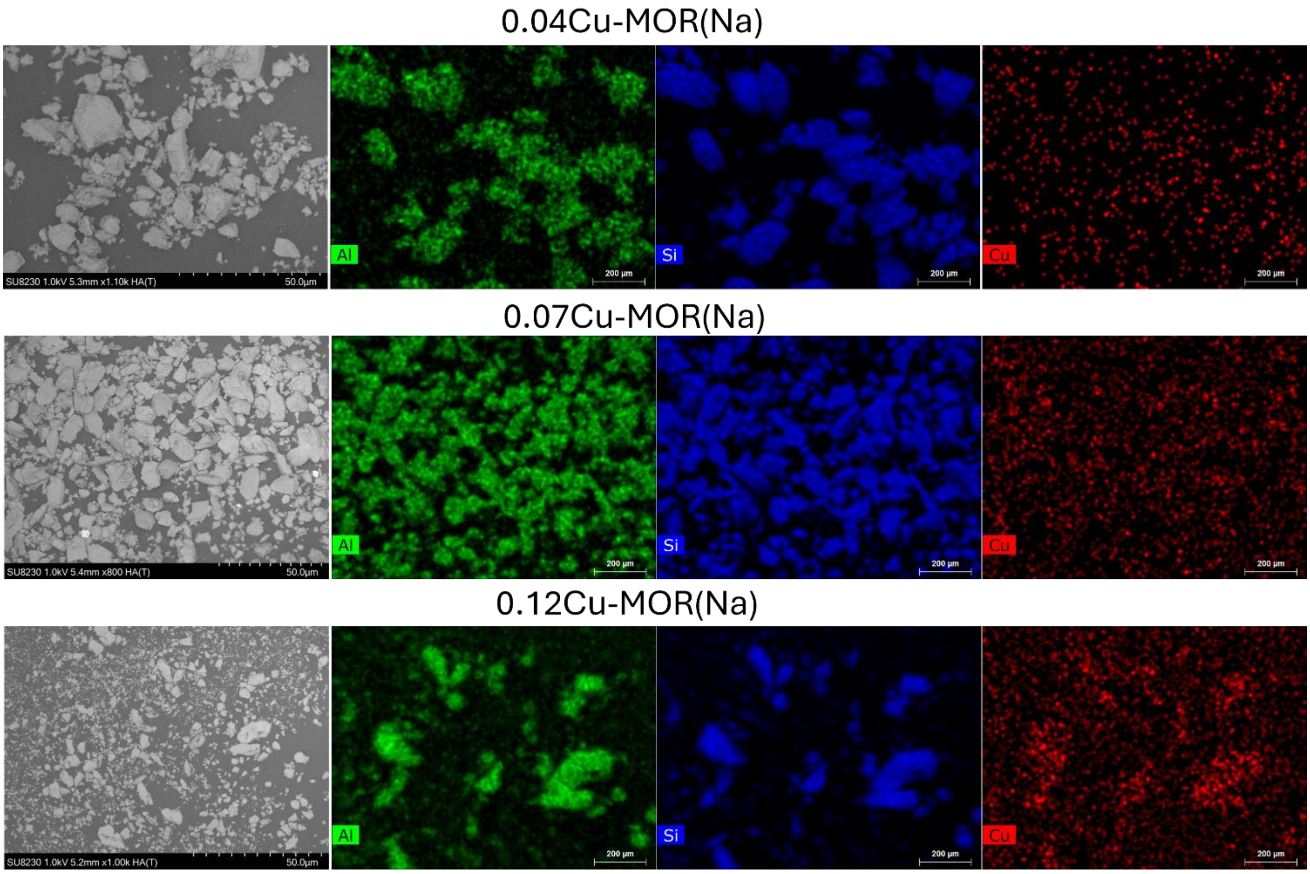

Figure S12: Backscattering and EDX images of the materials with different Cu/Al ratio.

12.References

1. Höfer P, Grupp A, Nebenführ H, Mehring M. Hyperfine sublevel correlation (hyscore) spectroscopy: a 2D ESR investigation of the squaric acid radical. *Chemical Physics Letters*. 1986;132(3):279-282. doi:10.1016/0009-2614(86)80124-5

2. Stoll S, Schweiger A. EasySpin, a comprehensive software package for spectral simulation and analysis in EPR. *Journal of Magnetic Resonance*. 2006;178(1):42-55. doi:10.1016/j.jmr.2005.08.013

3. Wijerathne A, Sawyer A, Daya R, Paolucci C. Competition between Mononuclear and Binuclear Copper Sites across Different Zeolite Topologies. *JACS Au*. 2024;4(1):197-215. doi:10.1021/jacsau.3c00632

4. Guo J, Sours T, Holton S, Sun C, Kulkarni AR. Screening Cu-Zeolites for Methane Activation Using Curriculum-Based Training. *ACS Catal*. 2024;14(3):1232-1242. doi:10.1021/acscatal.3c05275
